# Comprehensive Review: Technological Approaches, Properties, and Applications of Pure and Reinforced Polyamide 6 (PA6) and Polyamide 12 (PA12) Composite Materials

**DOI:** 10.3390/polym17040442

**Published:** 2025-02-08

**Authors:** Marcel Kohutiar, Lucia Kakošová, Michal Krbata, Róbert Janík, Jozef Jaroslav Fekiač, Alena Breznická, Maroš Eckert, Pavol Mikuš, Ľudmila Timárová

**Affiliations:** 1 Faculty of Special Technology, Alexander Dubcek University of Trenčín, Ku Kyselke 469, 911 06 Trenčín, Slovakia; lucia.kakosova@tnuni.sk (L.K.); michal.krbata@tnuni.sk (M.K.); jozef.fekiac@tnuni.sk (J.J.F.); alena.breznicka@tnuni.sk (A.B.); maros.eckert@tnuni.sk (M.E.); pavol.mikus@tnuni.sk (P.M.); ludmila.timarova@tnuni.sk (Ľ.T.); 2 Faculty of Industrial Technologies in Púchov, Alexander Dubček University of Trenčín, Ivana Krasku 491/30, 020 01 Púchov, Slovakia; robert.janik@tnuni.sk

**Keywords:** PA6, PA12, mechanical properties, material properties, structure

## Abstract

This article presents a comprehensive analysis of polyamide 6 (PA6) and polyamide 12 (PA12) composites fabricated using additive manufacturing technologies such as Selective Laser Sintering (SLS) and Multi Jet Fusion (MJF). It focuses on the mechanical properties, preparation processes, and the influence of technological parameters on the final material characteristics. PA6 is characterized by a higher degree of crystallinity, contributing to its strength and resistance to high temperatures, whereas PA12 exhibits a more amorphous structure, offering better dimensional stability and lower moisture absorption. The article examines these properties and their implications for the use of composites in various applications. Applications of PA6 and PA12 composites span a wide range of industries, including automotive, aerospace, and electronics, where they provide a combination of high strength, wear resistance, and chemical stability. Mechanical properties, such as tensile strength and toughness, are analyzed within the context of modern manufacturing processes, with MJF technology delivering more homogeneous properties compared to traditional methods. The preparation process of these composites involves optimizing temperature, cooling rates, and material layering, which significantly impact the final properties and the applicability of the composites.

## 1. Introduction

Additive manufacturing (AM), also known as 3D printing, represents a revolutionary approach to part production, enabling the realization of complex geometric structures through the layer-by-layer deposition of material [1,2]. Unlike traditional manufacturing methods based on material removal or shaping, this process minimizes waste, reduces production time, and enhances design flexibility. AM has gained significant importance in industries such as automotive, aerospace, and biomedicine due to its ability to produce highly precise components with customized properties [1,2].

Polyamides, particularly PA6 and PA12, are among the key materials utilized in additive manufacturing [1]. Their popularity stems from their excellent mechanical properties, chemical resistance, and adaptability to various technological processes. PA6 is characterized by high strength, stiffness, and wear resistance, making it an ideal material for load-bearing structures and applications in the automotive and electronics sectors [3,4]. Conversely, PA12 excels in thermal stability, low porosity, and manufacturing flexibility, enabling its extensive use in aerospace and biomedical fields [5].

Polyamide 6 (PA6) and polyamide 12 (PA12) are known for their excellent mechanical properties, which are closely linked to their structure. PA6 has the ability to form polymorphic crystalline modifications, such as γ- and α-phases, with distinct melting temperatures (208 °C for γ and 278 °C for α-modification). These structural differences significantly influence the material’s strength and stiffness. Processing conditions, such as cooling rates or drawing, play a crucial role in forming these modifications, allowing for targeted customization of PA6 properties. The material exhibits high strength but is more susceptible to moisture absorption compared to PA12 [6,7].

PA12, on the other hand, is characterized by longer carbon chains, which reduce the density of hydrogen bonds, increasing its flexibility and resistance to moisture. Its homogeneous structure contributes to its excellent mechanical properties, such as strength and wear resistance. PA12 exhibits anisotropic behavior, with properties like modulus of elasticity and hardness significantly influenced by the orientation of layers during processing. For example, vertically oriented samples achieve up to 50% higher reduced Young’s modulus and superior wear resistance compared to horizontally oriented samples [7].

The chemical stability of PA12 supports its compatibility with fillers, such as glass fibers, which further enhance its strength and load resistance. These properties make PA12 an ideal material for applications requiring high strength, flexibility, and moisture resistance [7].

A critical feature of these polymers is their ability to be reinforced with materials such as carbon or glass fibers. Research indicates that the use of a PA12 matrix in combination with carbon fibers results in higher resistance and potentially lower moisture absorption compared to PA6, thereby enhancing the long-term durability of components in extreme conditions [8]. These additives enhance mechanical properties such as tensile strength, elastic modulus, and creep resistance, thus paving the way for applications in extreme conditions [9,10]. Modern technologies, such as Multi Jet Fusion (MJF) and Selective Laser Sintering (SLS), allow for the efficient integration of these fillers, resulting in components with high precision and minimal porosity [11].

Another technology is Selective Absorption Fusion (SAF), which is suitable for a wide range of industrial sectors and applications. Three-dimensional printing with SAF utilizes industrial printheads to deposit a binding agent onto the surface of powder materials. Subsequent exposure to an infrared lamp ensures the bonding of particles layer by layer, with the agent enhancing the ability of powder particles to absorb heat, leading to the formation of solid objects. Materials such as Nylon 11 (PA11), Nylon 12 (PA12), and Polypropylene (PP) are commonly used [12].

On the other hand, PA6-based processes offer greater flexibility in the development of glass fiber-reinforced composites, are more cost-effective, and provide resistance to dynamic loading. These properties make PA6 well-suited for applications where high mechanical strength at lower costs is essential [13].

Processing parameters, including processing temperature, cooling rate, and layer orientation, fundamentally influence the final properties of the parts. In 3D printing, particularly with MJF and SLS technologies, anisotropy resulting from material layering can affect part strength in different directions, presenting challenges in optimizing mechanical properties [3,5].

Additionally, PA6 and PA12 enable the production of components with complex geometries that would be difficult or impossible to achieve with traditional methods, such as injection molding or compression molding. PA6 is frequently employed in the manufacture of components resistant to mechanical stress, such as gears, bearings, and engine covers [3]. PA12, on the other hand, is ideal for applications requiring high precision and low porosity, such as medical devices or aerospace components [5]. Furthermore, PA12’s properties make it suitable for applications demanding resistance to chemicals and moisture, enhancing its value in specific industrial sectors [10].

In the context of additive manufacturing, it is crucial to emphasize that optimizing processing parameters and selecting appropriate reinforcements can significantly affect composite performance. Glass fibers provide excellent impact resistance and wear resistance, while carbon fibers contribute to increased strength and thermal stability [9]. These attributes enable PA6 and PA12 to compete with traditional materials in demanding industrial applications, such as automotive and aerospace industries [10].

The combination of their chemical and physical properties with the ability to form composites offers the potential to design highly specialized components that enhance performance and efficiency in manufacturing processes. This study, therefore, emphasizes the pursuit of optimal utilization of PA6 and PA12 in the context of modern technological advancements, their development, and their implementation in industrial applications [10,11].

The aim of the publication is to explore the potential of polyamides PA6 and PA12 in additive manufacturing with an emphasis on their physical and chemical properties, reinforcement options and their broad industrial applications. Special attention is paid to the comparison of reinforced variants of PA6 and PA12 containing carbon or glass fibers. These reinforcements significantly improve their mechanical properties, such as tensile strength, elastic modulus, wear resistance and thermomechanical stability. The publication analyzes the various mechanical properties of these reinforced materials, with emphasis on their anisotropy caused by layering during additive manufacturing. Differences in strength, toughness and temperature resistance are also investigated, which allows the evalution of the suitability of these materials for specific industrial applications.

## 2. Polyamide 6

Polyamide 6 (PA6) is a versatile engineering polymer that, due to its chemical and physical structure, offers a wide range of mechanical, thermal, and tribological properties. PA6 is characterized by high tensile strength, toughness, excellent wear resistance, and impact energy absorption capability. These attributes make it suitable for use in various industrial sectors, including automotive, electrical, and packaging industries [14,15]. Within both additive manufacturing and conventional processing methods, PA6 facilitates the development of composite materials, which, due to their adaptability, find applications in demanding scenarios [16,17].

PA6 is a semi-crystalline polymer that provides an ideal balance between stiffness and flexibility. The crystallinity of PA6 can be modulated through various processing techniques, influencing its glass transition temperature, thermal stability, and other material properties [5,6]. Recent advancements have emphasized the incorporation of reinforcing fillers such as carbon nanotubes, glass fibers, and wollastonite. These additives significantly enhance the performance characteristics of PA6, imparting greater stiffness, thermal resistance, and reduced wear to the composites [18,19].

Fillers such as glass fibers and carbon nanotubes form bonds with the PA6 matrix through polar amide linkages, enabling adhesion to the functional surface of the fillers. This enhances the mechanical properties of the composites, including tensile strength, elastic modulus, and resistance to temperature fluctuations. Additionally, the use of surface treatments on fillers further improves compatibility between the fillers and the matrix [18,19].

### 2.1. Characteristics and Applications of PA6

PA6 is inherently suited for the development of composite materials due to its ability to form strong bonds with various reinforcing materials. The chemical structure of PA6 includes polar amide bonds, which enable interactions with functional filler surfaces. This characteristic is crucial in designing composites with optimized mechanical properties. For instance, carbon nanotubes or oil-derived nanofillers can enhance thermal degradation resistance and improve load-bearing strength [14,16,18].

PA6 is resistant to chemicals due to its structure and ability to form various polymorphic crystalline modifications. These modifications, particularly the α-phase with a higher melting temperature (278 °C), are characterized by high crystallinity, which limits the penetration of chemicals into the material. Additionally, the structural regularity and dense arrangement of molecules prevent the diffusion of chemicals.

The article suggests that PA6 can alter its crystalline structure under different conditions, which can impact its chemical stability. For instance, cooling from the melt results in the formation of the γ-modification, while higher temperatures lead to the more resistant α-modification. These crystalline structures enable the material to resist chemical attacks by minimizing the interaction between chemicals and the polymer molecules.

In this way, PA6 not only achieves high mechanical strength but also excellent resistance to chemical substances [6].

The addition of carbon nanotubes (CNTs) and long carbon fibers (LCFs) to the PA6 matrix not only enhances its mechanical properties, such as strength and elastic modulus, but also reduces water absorption, a characteristic challenge for PA6. CNT and LCF create a barrier structure that limits water penetration into the matrix, thereby extending the material’s lifespan in demanding conditions where moisture poses a risk. Research also indicates that these fillers improve the thermal stability of PA6 and minimize dimensional changes associated with water absorption, which is critical for applications requiring high long-term durability, such as automotive components or construction elements.

By embracing these material innovations, it is possible to optimize the properties of PA6 for applications where traditional unmodified polyamides fail due to higher susceptibility to property degradation from moisture and time. This opens new opportunities for the engineering use of PA6 in highly demanding environments [20,21,22,23].

PA6 composites find applications not only in technically demanding scenarios but also in everyday products. In the automotive industry, they are used for manufacturing engine components, pumps, and fuel tanks, while in electronics, they serve as the foundation for housings and insulators. Their resistance to chemicals and excellent electrical properties make them a preferred material for complex engineering applications [19,24].

### 2.2. Mechanical Properties of PA6

#### 2.2.1. Mechanical Durability, Dynamic Properties, and Impact Toughness and the Effect of Temperature on Mechanical Properties

**Figure 1:**
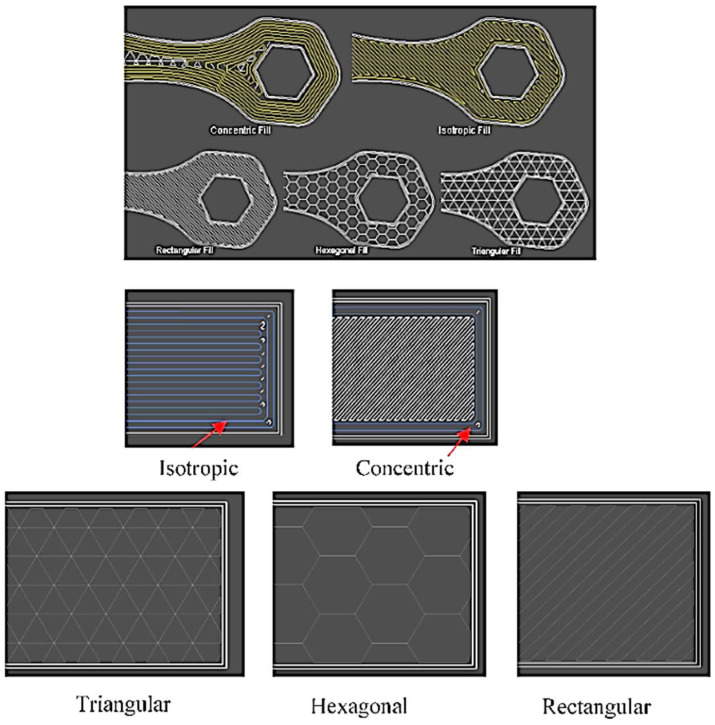
Graphical description of fiber distribution and matrix options in Eiger^®^ X3 software [18].

Mechanical properties also vary significantly depending on the manufacturing technology used. In additive manufacturing, such as 3D printing, anisotropy in mechanical properties often occurs due to the layered structure of the material, as highlighted by Díaz-Rodríguez et al. in their study (Figure 1) [18]. However, in traditional manufacturing methods like injection molding, properties are more homogeneous and predictable [24].

Dynamic loading and fatigue are critical factors affecting the lifespan of PA6 composites in applications where components are subjected to cyclic. Research indicates that reinforcement significantly reduces susceptibility to fatigue failure. For instance, glass beads in PA6 enhance fatigue resistance by evenly distributing stress within the matrix [17,25].

**Figure 2:**
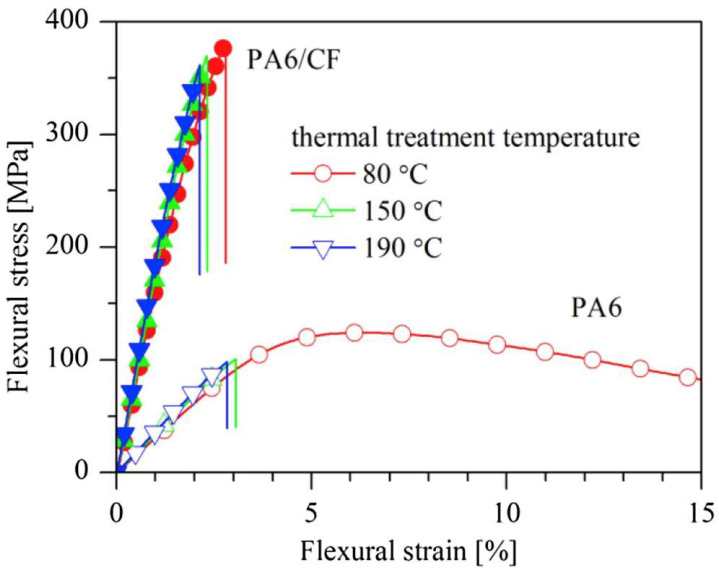
Typical stress-strain curves for PA6 and PA6/CF composites with various thermal annealing conditions. Reproduced with permission from [23], Elsevier, 2025.

During dynamic load testing, a decrease in mechanical response was observed at higher temperatures, which is typical for thermoplastic polymers. However, PA6 composites retain their properties better than pure PA6 (see Figure 2), attributed to the strengthening of interfacial bonds between the matrix and the filler [16,19].

#### 2.2.2. Impact Toughness and the Influence of Temperature on Mechanical Properties

The impact toughness of PA6 composites is a critical property, making them suitable for applications involving impact loading. Testing has demonstrated that composites reinforced with high-temperature glass fibers (HSHTs) exhibit superior performance compared to those reinforced with carbon fibers, attributed to the better compatibility of glass fibers with the PA6 matrix [15,25].

Temperature significantly influences impact toughness. At temperatures below the glass transition point of PA6, the material becomes more brittle, while increased temperatures lead to improved toughness up to a certain threshold, after which the properties deteriorate due to matrix softening. These changes are closely tied to the thermodynamic properties of PA6, which exhibit a pronounced dependence on temperature, as noted by Paolucci et al. [19].

### 2.3. Production and Characterization of PA6 Material

The production of PA6 material encompasses various technological approaches, each with specific characteristics that influence the final material properties. Commonly used methods include injection molding, compression molding, 3D printing, and extrusion [14,17,18,19].

Injection molding is one of the most prevalent methods for manufacturing PA6. This process enables the rapid production of precise parts with tight tolerances. Its advantage lies in the uniform distribution of fillers; however, the resulting properties can be affected by fiber orientation during material flow within the mold [14,17].

Compression molding is primarily utilized for producing large components. This process provides a high degree of homogeneity and allows for the incorporation of high filler or reinforcement content, enhancing the mechanical properties of composites. However, its disadvantage is a longer production time [16,24].

**Figure 3:**
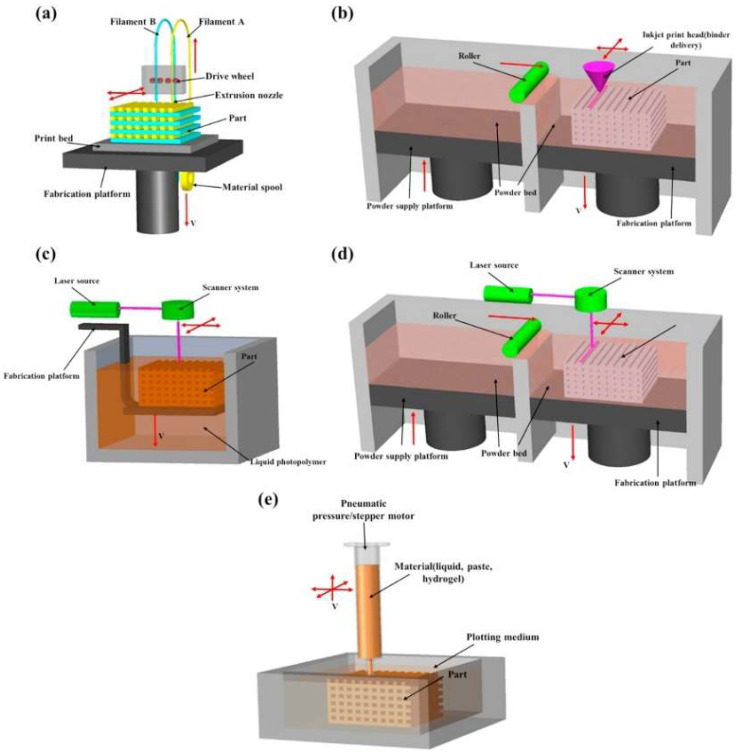
Schematic representation of a typical systems: (**a**) FDM system; (**b**) 3DP system; (**c**) SLA system; (**d**) SLS system; (**e**) 3D drawing system. Reproduced with permission from [25], Elsevier, 2025.

Three-dimensional printing, specifically methods such as Fused Deposition Modelling (FDM) (Figure 3), Selective Laser Sintering (SLS), and Multi Jet Fusion (MJF), offers significant flexibility in material production. These technologies facilitate the creation of parts with complex geometries, but they are limited by porosity and lower reproducibility of properties compared to traditional methods [17,18].

Additionally, methods like MJF and FDM support homogeneous filler distribution, resulting in improved isotropic properties and better reproducibility of outcomes [16,17]. When fillers such as carbon fibers are used, these composites exhibit enhanced impact resistance, wear resistance, and tolerance to temperature variations [15,17].

Extrusion is suitable for the production of profiles or materials in the form of fibers and granules. This process is ideal for continuous production, enabling efficient dispersion of fillers and reinforcements [25].

The characterization of PA6 material is essential for understanding its structure and behavior under various conditions. Fundamental methods include thermal analysis, mechanical testing, and microscopic techniques [15].

Mechanical testing encompasses tensile, compressive, and flexural tests, which evaluate strength, elasticity, and resistance to deformation. These tests have revealed that PA6 composites with various reinforcements exhibit increased strength and stiffness, with glass beads and wollastonite being particularly effective in improving wear resistance [14,18].

**Figure 4:**
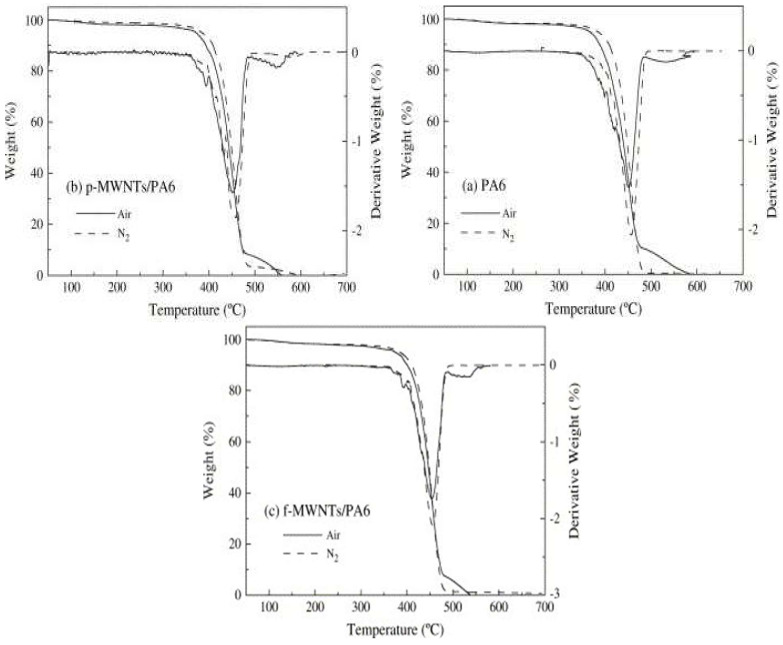
TGA and DTG curves: (**a**) PA6; (**b**) p-MWNTs/PA6; (**c**) f-MWNTs/PA6 composites with 1.0 wt.% MWNTs in nitrogen and air atmospheres [14].

In Figure 4, thermogravimetric analysis (TGA) was used to investigate the thermal stability of pure polyamide 6 (PA6) and its multi-walled carbon nanotube (MWNT) reinforced composites in air and nitrogen environments. The results indicate that the environment significantly affects the degradation behavior of all samples. Pure PA6: Figure 4a shows that PA6 exhibits rapid weight loss at temperatures above 400 °C, with air causing more intense degradation compared to nitrogen.PA6 with p-MWNT: The sample with added crude MWNTs (Figure 4b) shows a slight improvement in thermal stability in nitrogen environment, while degradation in air proceeds similarly to that of pure PA6.PA6 with f-MWNTs: Functionally modified MWNTs (Figure 4c) contribute to a significant increase in thermal stability in both environments. The modification of MWNTs likely improves the matrix-filler interaction, thereby retarding thermal degradation. These results confirm that the modification of carbon nanotubes can significantly increase the thermal stability of PA6 composites, which is crucial for their use in harsh environments [14].

**Figure 5:**
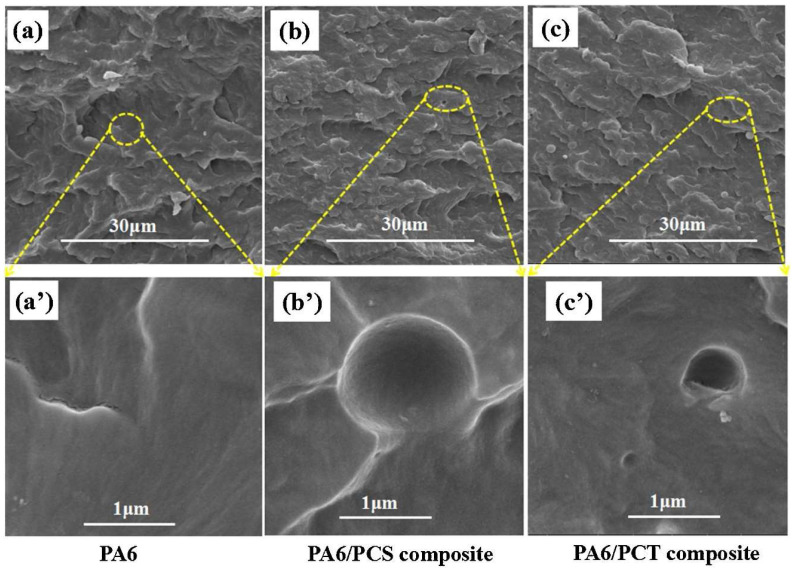
(**a**) Images show the morphology of pure PA6; (**b**) PA6/PCS composite; (**c**) PA6/PCT composite, where the holes on the surface indicate the uniform dispersion of PCS and PCT after etching with tetrahydrofuran, with PA6/PCT exhibiting better interaction between the filler and the matrix. In detail, (**a’**) reveals small cracks that highlight the brittle nature of the pure PA6 fracture, (**b’**) shows a distinct cavity left by the filler particle, influencing the composite’s mechanical response, and (**c’**) displays a smaller cavity, suggesting improved interaction between the PCT filler and the PA6 matrix [16].

Microscopic analyses, such as Scanning Electron Microscopy (SEM), are used to evaluate the microstructure and filler distribution within the matrix. These methods have revealed that uniform filler distribution is crucial for achieving optimal mechanical properties (see Figure 5) [25,26].

Process parameters such as temperature, pressure, and cooling rate significantly influence the final properties of PA6 composites. For instance, rapid cooling can result in a higher crystalline fraction, which enhances strength but may reduce the material’s toughness [15,17].

Anisotropy is a common issue, particularly in 3D printing and injection molding. Proper alignment of reinforcements and control of material flow can minimize these adverse effects and improve property homogeneity [18,24]. Optimizing process parameters, such as extrusion or molding speed, can lead to significant improvements in the mechanical and thermal properties of the composites [18,25].

Polyamide 6 (PA6) is characterized by its challenging melt processing behavior, primarily due to the sudden drop in viscosity, which can significantly impact the quality of the final product. This phenomenon necessitates the use of specialized extrusion screws with a very short compression zone, ensuring uniform material distribution and minimizing potential defects during processing. Proper temperature settings and optimization of the screw geometry are critical to maintaining process stability and achieving the desired properties of PA6. These specific processing requirements enhance the utility of PA6 in additive manufacturing and expand its application potential in various industrial sectors [12].

### 2.4. Applications and Challenges of PA6 Material in Industry

PA6 materials find wide applications in various industrial sectors due to their exceptional combination of mechanical, thermal, and tribological properties. This material is particularly attractive for the automotive, aerospace, construction, and electronics industries, where high strength, low weight, and wear resistance play a key role [16,24].

The aerospace industry benefits from the low density and excellent mechanical durability of these materials, which allows for weight reduction in aircraft and improved energy efficiency. PA6 composites are used, for example, in the production of interior panels and smaller structural components [16,25].

In electronics, these composites are utilized in the manufacture of components resistant to moisture and temperature fluctuations, such as housings for electrical devices and insulation materials. In construction, they are used for pipes, reinforcement elements, and panels, with advantages including wear resistance and long service life [17,19].

Despite their many advantages, the production and application of PA6 composites face several challenges. One major issue is the anisotropy of mechanical properties, which arises during the manufacturing process, especially in additive methods such as 3D printing. This anisotropy can negatively affect the performance of composites depending on the load orientation [16,18].

Another challenge is the optimization of processing parameters during production. Achieving the desired properties requires proper control of temperature, pressure, and processing time, as each of these factors can significantly influence the final properties of the composite, such as its mechanical strength, thermal stability, and moisture resistance [17,25]. PA6 composites are commonly used for components such as housings, bearings, and parts for the automotive and electronic industries, where high strength and chemical resistance are required [17,25].

Additionally, considerable attention is given to the recycling of PA6 composites, as these materials often contain various fillers and reinforcements that complicate their reprocessing. The development of efficient recycling methods is necessary to enhance sustainability and reduce environmental impact [19,24].

Finally, improving adhesion between the matrix and reinforcements is necessary, which can be achieved through surface modification of reinforcements or the use of specialized coupling agents. This aspect is critical for improving interfacial properties, which significantly affect the overall performance of composites [15,18].

## 3. Polyamide 6 Reinforced with Carbon Fiber

Polyamide-6 (PA6) represents one of the most significant thermoplastics used in industrial applications, thanks to its exceptional strength, stiffness, and chemical resistance. Its properties make it suitable for use in combination with various types of reinforcing materials, significantly enhancing its functional characteristics. The incorporation of carbon fibers (CF) into the PA6 matrix is a key technology that provides additional mechanical and thermal advantages, as well as increased wear resistance, opening new possibilities for the use of these composites in highly demanding applications [20,26].

**Figure 6:**
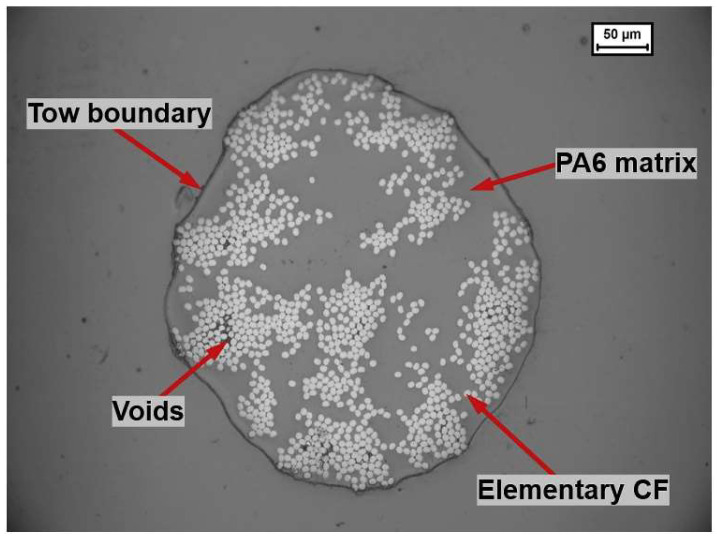
Typical cross-section of a CF/PA6 fiber visualized using optical microscopy. Reproduced with permission from [26], Elsevier, 2025.

In recent years, research has focused on optimizing the processing of CF/PA6 composites. Key manufacturing techniques include melt processing and additive technologies, which enable precise control over the volume fraction of carbon fibers and the elimination of microvoids. Research findings suggest that adding carbon fibers to PA6 can increase tensile strength and elastic modulus by 50–60%, while slightly reducing elongation at break. These properties make CF/PA6 composites an ideal material for applications (Figure 6) where a combination of low weight and high strength is crucial, such as in the automotive and aerospace industries [21,22].

Designing and manufacturing composites with continuous or short carbon fibers poses challenges related to the fiber-polymer matrix interface. Successful dispersion and adhesion of fibers to the matrix significantly influence their mechanical properties. Efforts in this field are focused on applying surface treatments to carbon fibers, which improve the interface and minimize the risk of fiber separation under load [23,27].

In addition to mechanical advantages, CF/PA6 composites also exhibit remarkable thermal properties. Studies indicate that carbon fibers promote the formation of the α-phase of PA6 crystals, enhancing the composite’s crystallinity and thermal stability. This characteristic is critical for applications requiring resistance to high temperatures and thermal shocks [22,28].

The integration of recycled carbon fibers into PA6 represents another promising direction that could reduce the environmental footprint of industrial composites. Louis Jeantet et al. highlight that recycled carbon fibers (RCFs) have demonstrated comparable mechanical properties to virgin fibers, while their lower cost and ecological benefits make them an attractive solution for sustainable manufacturing processes [29].

PA6 reinforced with carbon fibers thus emerges as a strategic material for the future of industrial production, combining excellent mechanical properties with the potential for ecological innovation. Further research and technological advancements, as noted by Louis Jeantet et al., open new opportunities for its broader application across various industries [29].

### 3.1. Production of Carbon-Fiber-Reinforced PA6 Composites

**Figure 7:**
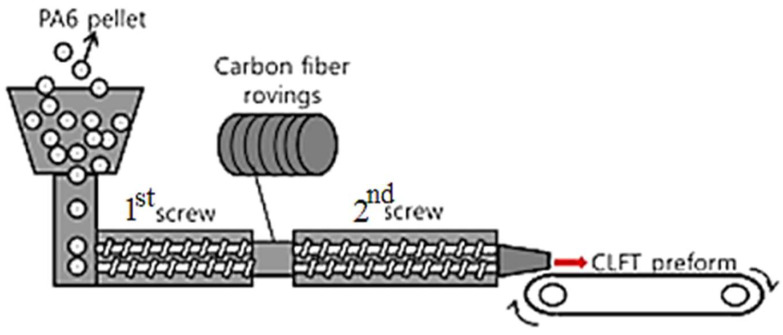
Manufacturing process of PA6/LCF composites. Reproduced with permission from [21], Springer Nature, 2025.

One of the most widely used techniques is melt extrusion, where carbon fibers are continuously embedded into the PA6 matrix during the melting process (Figure 7). This method ensures uniform fiber distribution within the matrix, achieving high interfacial adhesion between the fibers and the polymer. It has been found that adding carbon fibers to PA6 increases the elastic modulus and tensile strength, while reducing elongation at break, indicating an increase in material stiffness [20,21].

Three-dimensional printing technologies, such as Fused Deposition Modeling (FDM), offer new possibilities for producing composites reinforced with continuous carbon fibers. FDM allows precise control over fiber orientation, which significantly affects mechanical properties. However, one challenge of this method is the formation of microscopic pores that reduce composite strength. Research has shown that post-printing processes, such as pressure molding, can reduce pore content and improve interfacial properties, resulting in better stress resistance [22,27].

Impregnation technologies involve infiltrating molten PA6 oligomer into fibers, which are subsequently polymerized. This technology enables achieving a high fiber content in the composite, up to 60 wt.%, significantly enhancing the material’s strength and stiffness. Composites prepared this way exhibit high thermal stability, with deformation temperatures approaching the melting point of PA6 [23,28].

Innovative processes, such as those involving nanotechnology, include adding multi-walled carbon nanotubes (MWCNTs) or graphene oxide (GO) to the PA6 matrix. These fillers improve PA6 crystallization and act as nucleating agents, enhancing thermal stability and mechanical properties. Additionally, mechanical mixing of nanotubes with PA6 ensures uniform filler distribution, which helps reduce the coefficient of friction and wear [27,29].

Advantages and Disadvantages of PA6 Composite Preparation Processes [20,21,22,27]:

1. Melt Extrusion:
  - Advantages: Uniform distribution of carbon fibers within the matrix, strong interfacial adhesion between fibers and polymer, improved mechanical properties (tensile strength, elastic modulus), and thermal stability.
  - Disadvantages: Higher likelihood of microscopic pore formation and limited ability to control fiber orientation in traditional setups.
2. Fused Deposition Modeling (FDM):
  - Advantages: Precise control of fiber orientation, capability to produce complex geometries and, reduction in manufacturing costs.
  - Disadvantages: Pore formation, lower composite strength compared to other methods, and the need for post-processing (e.g., compression molding) to enhance properties.
3. Impregnation and Subsequent Polymerization:
  - Advantages: High fiber content (up to 60% by weight), increased material strength and stiffness and, homogeneous crystalline structure.
  - Disadvantages: Higher equipment costs and longer preparation time.
4. Nanotechnology (MWCNT, Graphene Oxide):
  - Advantages: Improved PA6 crystallization, reduced water absorption, enhanced thermal stability, and wear reduction.
  - Disadvantages: Higher material costs and technical challenges in achieving homogeneous distribution of nanomaterials.

Modern methods (FDM, nanotechnology, and advanced impregnation techniques) enable more precise control over material properties, better compatibility between fibers and the matrix, and enhanced mechanical and thermal characteristics. However, they require more advanced equipment and longer production times. Traditional methods (melt extrusion and injection molding) are less expensive and suitable for high-volume production but have limited capabilities in controlling the composite microstructure [20,21,22,27].

### 3.2. Properties and Structure of Carbon-Fiber-Reinforced PA6 Composites

Polyamide-6 (PA6) reinforced with carbon fibers (CFs) belongs to the category of composites with excellent mechanical and thermal properties, making them suitable for demanding applications. The mechanical properties of PA6/CF composites result from the synergy between the matrix, carbon fibers, and their interfacial interactions. Adding CF increases tensile strength by approximately 60% and the elastic modulus by 50%, while dynamic mechanical analysis reveals a storage modulus improvement of over 45% compared to pure PA6 [20,21,23]. The loss modulus of these composites is higher, indicating their ability to transfer energy more efficiently. Jerzy Myalski et al. note that the increased stiffness of the material also leads to a reduction in elongation at break, indicating higher brittleness of the composite [27].

A critical area of research on PA6/CF composites is their crystalline structure and the influence of CF on the crystallization process, as described by Xinlei Yan et al. CFs act as nucleating agents that promote the formation of the crystalline α-phase while reducing the size of spherulites. These changes improve the material’s resistance to crack initiation and contribute to higher interfacial strength. The transcrystalline layer formed around the CF plays a significant role in enhancing mechanical and thermal properties [20,27,28]. Furthermore, functionalization of CFs through surface treatment improves compatibility with PA6 and facilitates even more efficient crystallite formation [23].

**Figure 8:**
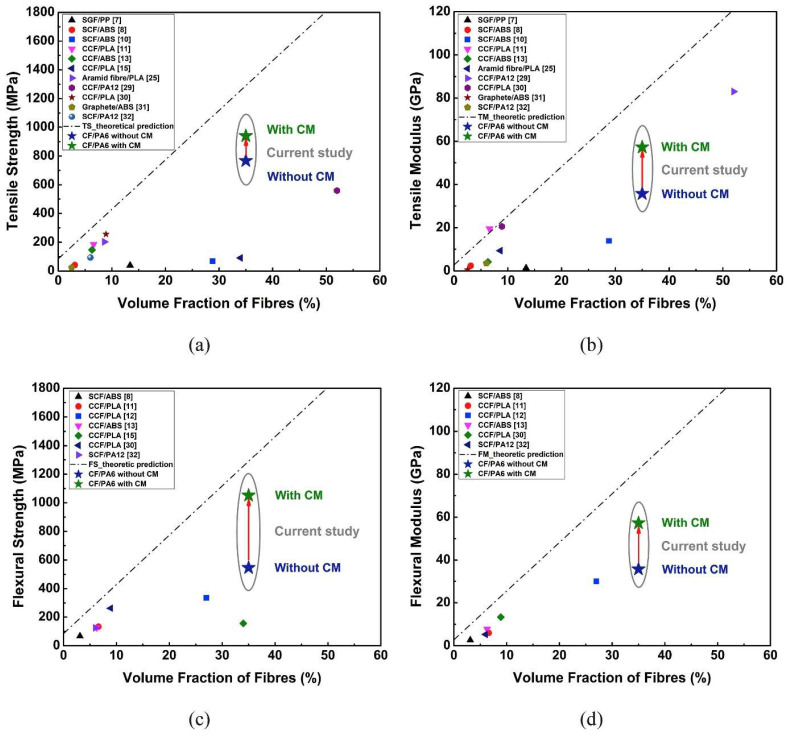
Mechanical properties of CF/PA6 in this study compared to composites produced using FDM and predictions based on the rule of mixtures: (**a**,**b**): tensile properties, (**c**,**d**): flexural properties. Reproduced with permission from [26], Elsevier, 2025.

The presence of CF also affects the thermal properties of PA6 composites. The crystallization temperature (Tc) increases to approximately 180 °C, a significant improvement compared to the pure matrix. Similarly, the glass transition temperature (Tg) of PA6/CF composites was approximately 70 °C and was not affected by the presence or content of carbon fibers [20,21]. Xinlei Yan et al. found that this increased resistance results from better distribution and stability of carbon fibers in the matrix, ensuring more uniform load distribution (Figure 8) [23].

PA6CF composites combine the excellent properties of the PA6 matrix and carbon fibers, ensuring high durability. The thermal stability of the composites is enhanced due to the fibers, which degrade at elevated temperatures. Mechanical properties, such as tensile strength and elastic modulus, are improved, although higher stiffness results in reduced elongation at break.

Moisture resistance is increased by the barrier effect of the fibers, extending the material’s lifespan in high-humidity environments. Strong interfacial adhesion between the fibers and the matrix minimizes the risk of microcracks and fatigue failure. Composites with long carbon fibers (LCFs) offer better fatigue resistance compared to those with short fibers.

Nevertheless, PA6CF has its limitations, such as high brittleness and more demanding manufacturing processes. Despite these challenges, these composites exhibit exceptional properties, making them ideal for demanding industrial applications [20,21,22,27].

One of the critical issues with PA6/CF composites is porosity, which can reach up to 12% in 3D-printed materials. This porosity reduces mechanical properties, such as tensile and flexural strength, by up to 30%. However, techniques like compression molding (CM) can lower the pore content to 6% and significantly improve mechanical parameters—transverse tensile strength increases by 78%, and flexural strength by 93% [22,28].

Compared to other fillers, such as short carbon fibers or graphite particles, long and continuous CFs provide significantly better strength and stability. These properties make PA6/CF composites ideal for industrial applications requiring high load resistance, such as in the automotive and aerospace industries [21,30].

### 3.3. Thermal, Crystallization, and Tribological Properties of PA6 Composites

Polyamide 6 (PA6) is one of the most widely used thermoplastics in the industry, with its properties significantly enhanced by the addition of carbon fillers such as carbon nanotubes (CNTs), carbon black (CB), or continuous carbon fibers (CCFs). These fillers provide a combination of mechanical durability, thermal stability, and tribological advantages, enabling broad industrial applications, particularly in the automotive and aerospace sectors. However, the improvement of thermal, crystallization, and tribological properties of PA6 composites depends on the type, amount, and compatibility of the fillers with the polymer matrix [27,28,29].

#### 3.3.1. Thermal Properties

The incorporation of CNT and CB into the PA6 matrix significantly enhances the thermal stability of composites. These fillers increase the thermal decomposition temperature and improve the composite’s ability to resist thermal deformations. The improvement mechanism lies in the ability of carbon fillers to evenly distribute heat and reduce concentrations of thermal stress. Studies have shown that composites containing CNT exhibit higher thermal stability than those with CB, which is attributed to their superior thermal conductivity and larger surface area, enabling more efficient heat transfer [27,29,31].

The storage modulus (which represents the stiffness of a material under dynamic loading and its ability to return to its original shape) of PA6/LCF composites increases with the rising content of long carbon fibers (LCFs), improving the composite’s ability to resist deformation under dynamic stress. A high loss modulus indicates the viscous nature of the composite, allowing it to dissipate energy as heat during cyclic loading. As the temperature increases, the viscosity of the composites decreases, affecting their dynamic behavior at higher temperatures. These dynamic properties demonstrate how carbon fiber content and temperature conditions significantly influence the performance of composites under cyclic loading [21].

Thermogravimetric analysis (TGA) provides data on the thermal stability of materials and their degradation at different temperatures. For example, the addition of carbon nanotubes or other fillers has been shown to increase the onset temperature of PA6 degradation, thereby enhancing its thermal resistance (see Figure 4) [15,19]. Differential Scanning Calorimetry (DSC) allows for the investigation of crystallinity and glass transition temperature, which directly influence the material’s mechanical properties [16,24].

The addition of 10 wt.% PCT (P(N-(4-carboxyphenyl)maleimide-alt-triallyl isocyanurate)) to PA6 and subsequent irradiation leads to an increase in the crystallinity percentage (Xc) by 61.4%. This increase is the result of a solid-state interfacial reaction (SSIR) between PA6 and PCT, which forms a strong interface that restricts the movement and rotation of PA6 macromolecules, thereby improving the material’s heat resistance, thermal conductivity, and moisture resistance [16].

**Figure 9:**
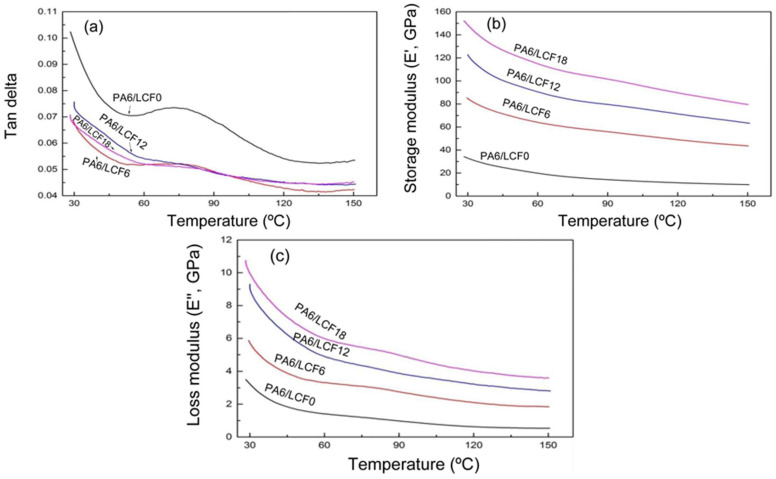
DMA test results of PA6/LCF composites: (**a**) Tan delta; (**b**) storage modulus; (**c**) loss modulus. Reproduced with permission from [21], Springer Nature, 2025.

The presence of continuous carbon fibers (CCFs) further enhances the ability of PA6 composites to withstand high temperatures. CCFs not only act as thermal reinforcement but also improve the material’s ability to maintain stability during rapid thermal changes. Tobias Donhauser et al. report that continuous carbon fibers in 3D-printed structures allowed for a 50% increase in the heat deflection temperature (HDT) compared to pure PA6, which is crucial for applications in high-temperature environments (Figure 9) [28].

#### 3.3.2. Crystallization Properties

In the area of PA6 crystallization, carbon fillers play a key role as nucleating agents. The addition of CNT leads to a reduction in spherulite radius and an increase in the crystallization rate, contributing to the formation of a finer and more homogeneous structure. Differential scanning calorimetry (DSC) analysis shows that the addition of 3 wt% CNT reduces the size of spherulites by 30%, resulting in a finer crystalline structure [27].

Functionalized CNTs further improve the interaction between the PA6 matrix and fillers, enabling more efficient polymer chain alignment and a more stable crystalline structure. This effect is particularly significant at lower temperatures, where CNTs reduce the activation energy required for crystallization, thus improving the processing efficiency [27,29].

Crystallization behavior is also observed with the addition of CCFs, which induce the formation of transcrystalline layers at the fiber-matrix interface. These layers not only improve the crystalline structure but also the mechanical integrity of the composites. X-ray diffraction (XRD) results confirmed an increase in the proportion of the α-phase crystals in PA6, leading to enhanced stiffness and strength [28,30].

#### 3.3.3. Tribological Properties

The tribological behavior of PA6 composites is one of the most important aspects for their practical use. The addition of carbon fillers, such as CNT and CB, significantly reduces the coefficient of friction and improves wear resistance. This effect is due to the lubricating action of carbon particles, which form a protective layer on the surface during friction. Composites with a combination of CNT and CB exhibit a synergistic effect that further reduces friction, but excessive filler concentration can reduce the overall cohesion of the material and increase surface roughness [23,30].

Fiber orientation in CCF composites plays a key role. Research has shown that parallel and antiparallel fiber orientations to the sliding direction result in lower wear, while normal orientation causes higher stress concentration at the fiber structure interfaces, leading to damage. Additionally, processes such as compression molding (CM) can reduce the void content in the material, increasing wear resistance and load-bearing capacity [27,28].

Thermal, crystallization, and tribological properties of PA6 composites are key parameters for their successful use in industrial applications. The introduction of carbon fillers and fibers brings significant improvements in these areas, with optimal results achieved through the correct combination of filler type, concentration, and processing method. Ongoing research in this field suggests that PA6 composites have great potential for further innovations in the area of high-performance materials [27,28,29,30].

### 3.4. Interfacial Properties: Adhesion Between Fibers and Matrix

The interfacial properties of composite materials, which include the adhesion between fibers and the matrix, are a critical factor influencing the mechanical performance, stiffness, and long-term stability of composites. In PA6 composites reinforced with carbon fibers, adhesion plays a key role in stress transfer between the fibers and the polymer matrix, maximizing the utilization of the high strength of carbon fibers. Optimizing adhesion requires not only surface modification of the fibers but also control over processing conditions and reduction in defect, such as porosity [32].

#### Influence of Fiber Surface Treatments (Sizing, CNT Functionalization)

**Figure 10:**
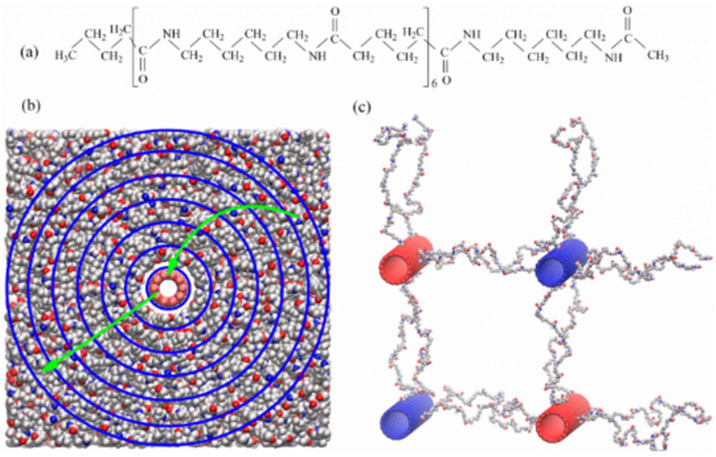
(**a**) Structure of PA-6,6 oligomers simulated in this study; (**b**) snapshot of a simulation box containing a single CNT (10, 0). Blue, red, gray, and white spheres represent N, O, C, and H atoms in the polymer matrix. The C atoms in the CNT, located at the center, are shown in red. Curved and straight arrows indicate the directions of unphysical and radial (physical) heat flow, respectively; (**c**) snapshot of a box containing 4 CNTs (17, 0) interconnected via PA-6,6 linkers. Only CNTs and linkers are shown for clarity. Heat transfer occurs from the cold (blue) CNTs to the hot (red) CNTs. All CNTs are oriented along the z-axis [22].

Fiber surface treatments, known as sizing, aim to improve the compatibility between carbon fibers and the PA6 matrix. In additive-manufactured composites, it has been demonstrated that using fibers with a special surface treatment leads to a dramatic improvement in the mechanical properties of the interface. Compared to virgin carbon fibers, fibers with sizing achieved a 42.2% increase in interlaminar shear strength (ILSS), resulting from better bonding between the fibers and the polymer matrix (Figure 10) [22,23].

The incorporation of carbon nanotubes (CNTs) into the polymer matrix represents another important approach to optimizing the interface. Daniele Zomer et al. emphasize that CNTs act as heterogeneous nucleating agents, and their functionalization improves compatibility with the PA6 matrix. For instance, the coupling of CNT with polymer chains not only reduces the Kapitza thermal resistance at the interface but also promotes the formation of a transcrystalline layer, which enhances the strength and wear resistance of the composites under mechanical stress [30].

Surface functionalization of CNT through grafting with 1,6-hexamethylene diamine (HMD) has been shown to be an effective method to increase compatibility between CNT and PA6. This technique leads to a reduction in the size of spherulites and improves their homogeneity, which is reflected in better mechanical properties of the resulting composites [32,33].

### 3.5. Interfacial Strengths Under Different Processing Conditions

Process conditions such as pressure, temperature, and porosity significantly influence interfacial strengths. Excessive molding pressure can lead to fiber damage and brittle failure, while low pressure results in insufficient bonding between the fibers and the matrix. Optimal pressure settings ensure uniform fiber impregnation and the formation of a stable interface, which significantly enhances the mechanical properties of the composites [23,27].

Porosity presents a key challenge in additively manufactured composites. Jerzy Myalski et al. report that high levels of porosity (up to 12%) weaken the mechanical properties of the composites. Findings indicate that reducing porosity through compression molding (CM—Compact Molding) leads to increases in tensile strength, flexural strength, and interlaminar fracture toughness by 78%, 93%, and 90%, respectively [27].

Processing temperature also affects the ability of fiber surfaces to interact with the matrix. Higher temperatures promote better fiber dispersion and the formation of a transcrystalline layer, which strengthens the interface and increases the deformation temperature of the composites. Analysis of local thermal properties shows that fibers with higher surface energy improve interfacial interactions and heat transfer between the matrix and the fibers [29,30].

#### Techniques for Improving Interfacial Properties

The use of polymer coupling agents and the application of fiber surface treatments are important techniques for optimizing interfaces. Studies by Xinlei Yan et al. have shown that proper application of surface treatment reduces the occurrence of fibers with insufficient adhesion, leading to a significant improvement in the strength of composites. Local thermal analysis (LTA) demonstrated that the presence of surface treatment increases the flexural deformation temperature by over 100% compared to untreated composites [23].

Functionalization of CNT led to an increase in ILSS and overall resistance to loading of the composites. Samples containing CNT with modified surfaces showed a 30% increase in strength and improved toughness, due to better stress distribution and fewer defects [33].

Adhesion between the fibers and the matrix is a fundamental prerequisite for achieving high-performance PA6 composites. Surface treatments such as sizing (fiber surface treatment) and CNT functionalization enhance compatibility and interfacial strength, while proper processing conditions minimize defects such as porosity. Advancements in these technologies open new possibilities for optimizing mechanical properties and applications of PA6 composites in demanding industrial conditions [22,33].

## 4. Polyamide 6 Reinforced with Glass Fiber

Polyamide 6 (PA6) is one of the most widely used engineering thermoplastics, extensively employed in the automotive and other industrial sectors due to its high strength, stiffness, and wear resistance. However, for applications that require even higher mechanical properties, such as tensile strength, stiffness, and wear resistance, PA6 is often reinforced with various filler materials, including glass fibers (GFs). These composites combine the properties of PA6 with the mechanical advantages of glass fibers, resulting in materials with excellent mechanical properties and stability at high temperatures [34].

PA6/GF composites are characterized by increased strength, stiffness, and impact resistance compared to unreinforced PA6. These materials are ideal for applications that require not only excellent mechanical strength but also long-term stability and wear resistance. Reinforcement with glass fibers enables the creation of materials with very high tensile strength and wear resistance [35]. Additionally, glass fibers provide PA6 composites with enhanced thermal stability and the ability to withstand aggressive chemicals and mechanical stresses, expanding their use in demanding industrial applications [36].

**Figure 11:**
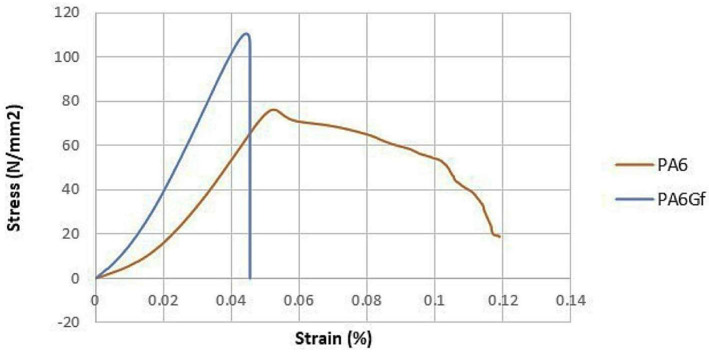
Stress-strain curve for fiber material PA6 compared to fiber material PA6GF [37].

Given the variety of processing methods, PA6/GF composites can be produced using various techniques, such as injection molding, extrusion, and newer technologies like D-LFT (Direct Long-Fiber Reinforced Thermoplastics), which allows for the efficient production of composites with long fibers. This process enables the manufacture of materials with uniform glass fiber distribution and optimal mechanical properties, ideal for various applications (Figure 11) [37]. The improvement in composite quality depends on proper processing, optimal temperatures, extrusion speeds, and fiber treatment, which enhance the adhesion between the matrix and fibers and ensure the desired properties of the composite [38,39].

### 4.1. Influence of Glass Fibers on the Mechanical Properties of PA6

#### 4.1.1. Increase in Strength, Stiffness, and Wear Resistance with the Use of Glass Fibers

Glass fibers have proven to be a highly effective reinforcement material for polyamide 6 (PA6) as they significantly improve its mechanical properties. Reinforcing PA6 with glass fibers leads to an increase in tensile strength, hardnessand, stiffness, as well as enhanced wear resistance. These properties are particularly beneficial for applications that require materials with high strength and wear resistance, such as automotive components, structural materials, or various mechanical parts. Glass fibers have the ability to evenly distribute the load within the composite, leading to a significant improvement in its lifespan. Experimental studies by Ke-qing Han, Zheng-jun Liu, and Mu-huo Yu have shown that PA6 composites reinforced with glass fibers exhibit excellent mechanical properties compared to pure PA6, especially when subjected to dynamic, static, and cyclic loads. This improved resistance is crucial, particularly in industries where materials are exposed to high mechanical stresses and abrasive wear [34].

#### 4.1.2. Influence of Glass Fiber Content on Tensile Strength, Flexural Strength, and Creep Resistance

One of the most significant effects of the presence of glass fibers in PA6 is the improvement of tensile strength and flexural strength. As the content of glass fibers in the composite increases, these mechanical properties also improve. Whitfield, T. et al. report that reinforced composites exhibit high tensile strength, which is crucial for applications where materials are subjected to tensile and flexural stresses, such as in the automotive and aerospace industries. Additionally, the presence of glass fibers significantly improves the material’s resistance to creeping (flow) under long-term loads, leading to better shape stability of the composite even at high temperatures. This effect is further intensified at higher glass fiber contents, which ensure better load distribution and minimize material deformation under long-term stress. On the other hand, at excessively high concentrations of glass fibers, processability may worsen, and the interaction between the fibers and matrix may be compromised, which could lead to weakening of the final mechanical properties. Research has shown that optimal glass fiber concentrations are around 30% to 40% by weight, which allows for excellent mechanical properties while maintaining good processability of the composite [35].

#### 4.1.3. Long-Term Mechanical Properties of PA6/GF Composites at Different Temperatures and Humidities

**Figure 12:**
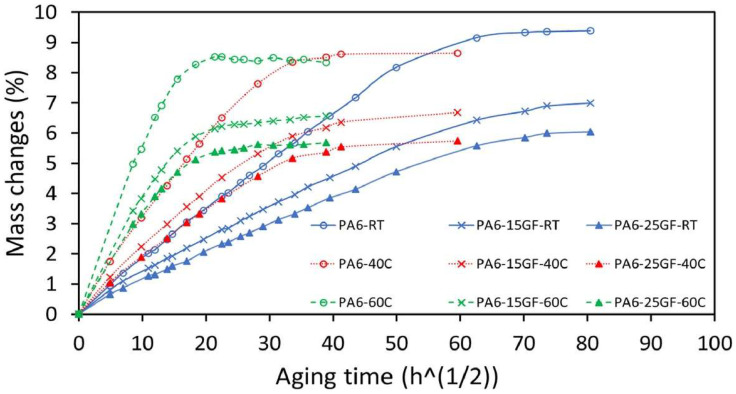
Water absorption curves for PA6 and its PA6-GF composites at room temperature (RT), 40 °C, and 60 °C. Reproduced with permission from [38], Elsevier, 2025.

The long-term mechanical properties of PA6/GF composites are strongly influenced by external conditions such as temperature and humidity. Prabhakaran, R. T. et al. report that moisture can significantly impact the mechanical properties of PA6, as water absorption (Figure 12) can lead to a deterioration in strength and stiffness. However, glass fibers, as a reinforcement, help reduce this absorption and stabilize the material’s mechanical properties under higher humidity. Glass fibers not only reduce the risk of water uptake into the matrix but also contribute to better shape stability under moisture and temperature cycles. PA6/GF composites exposed to various temperatures show better thermal resistance, with higher glass fiber content leading to better retention of mechanical properties even at elevated temperatures. On the other hand, long-term exposure to high temperatures may result in some degradation of the PA6 matrix, which can reduce the material’s mechanical properties, especially with prolonged heat exposure. Testing the composites under various temperature and humidity conditions revealed that glass fibers provide higher stability under long-term loads and improve the composite’s strength at higher operating temperatures [35].

#### 4.1.4. The Influence of Processing Temperature, Extrusion Speed, and Other Factors on the Quality of the Composite

Processing temperature and extrusion speed are key parameters that influence the production of PA6/GF composites. The extrusion temperature is crucial for the optimal melting of polyamide 6 (PA6), as it helps create a homogeneous melt that allows for uniform impregnation of glass fibers in the matrix. High processing temperatures ensure good melt fluidity, improving the distribution and orientation of the glass fibers in the matrix. This enhances mechanical properties such as tensile strength, flexural strength, and elastic modulus. However, excessive temperature can lead to polymer degradation and a reduction in the composite’s mechanical properties.

Extrusion speed, which determines the processing time of the melt, affects the uniformity and quality of fiber impregnation. At higher extrusion speeds, the quality of fiber distribution may decrease, leading to irregular structures and insufficient adhesion between the fibers and the matrix. For optimal processing of PA6/GF composites, it is important to set the extrusion temperature and extrusion speed to ensure the highest quality and mechanical properties of the material, with processing temperature balanced against extrusion speed and melt residence time in the process [34].

#### 4.1.5. Influence of Different Print Speeds and Temperature in 3D Printing of Composites

In 3D printing of PA6/GF composites, optimizing parameters such as print speed and extrusion temperature is crucial, as these factors directly influence the mechanical properties of the printed parts. Print speed determines how quickly the material is applied in individual layers, which can affect the homogeneity of the layers and their strength. At too high a print speed, fiber misalignment, pore formation, and a reduction in mechanical properties such as tensile strength and flexural strength can occur. On the other hand, at too low a print speed, the production time may increase, raising energy costs and potentially affecting surface quality.

Extrusion temperature is another critical factor, as it determines the flowability of the material during 3D printing. Too low an extrusion temperature can lead to poor adhesion between layers, resulting in weak bonding between the fibers and the matrix, thus reducing mechanical properties. Conversely, too high an extrusion temperature can cause polymer degradation, negatively affecting the quality of the composite. Research has shown that proper adjustment of these parameters, particularly extrusion temperature and print speed, leads to improved mechanical properties such as tensile strength, flexural strength, and wear resistance [35].

#### 4.1.6. Process Optimization for Improving Mechanical Properties

Optimizing the processing of PA6/GF composites is essential to achieve high mechanical properties required for specific industrial applications. One optimization method is adjusting the processing temperature, which affects the distribution of glass fibers in the matrix and their interaction with the polymer matrix. A properly chosen temperature ensures that the fibers are sufficiently impregnated, leading to better bonding between the fibers and the matrix. On the other hand, optimizing the extrusion speed ensures uniform fiber distribution, reducing the occurrence of defects such as pores or irregular fiber shapes that could weaken the composite’s mechanical properties. A significant factor is also optimizing the print speed in 3D printing, which ensures high-quality individual layers and proper fiber orientation. In fast printing, layer quality can decrease, reducing the strength and stability of the parts. By optimizing these parameters, improvements can be made in mechanical properties such as tensile strength, flexural strength, creep resistance, and stability at high temperatures and humidity levels. Research results have shown that properly set processing parameters lead to improvements in the overall mechanical and thermal insulation properties of PA6/GF composites, making them ideal for applications in the automotive, aerospace, and other demanding fields [36].

### 4.2. Applications of PA6/GF Composites in Industry

#### Applications in the Automotive and Aerospace Industries

PA6/GF composites, which combine the benefits of polyamide 6 (PA6) and glass fibers (GFs), have become a key material in the automotive and aerospace industries due to their excellent mechanical properties such as high tensile strength, stiffness, wear resistance, and low weight. In the automotive industry, these composites are used to manufacture various parts such as bumpers, panels, handles, and other components that must withstand high mechanical loads while being lightweight and cost-effective. Their high resistance to heat and mechanical wear, as well as their ability to resist chemicals and corrosion, make PA6/GF composites ideal for demanding applications in the automotive sector, where durability and long-term material stability under varying temperatures and humidity are required [34].

In the aerospace industry, PA6/GF composites are used to produce components that must meet strict requirements for strength, stiffness, and thermal properties at high operating temperatures. PA6/GF composites are ideal for applications where low weight is required without compromising mechanical properties. These composites are used in the production of covers, structural parts and instrument panel housings, as well as various engine components and other critical areas of the aircraft. Due to their excellent wear resistance, mechanical shock resistance, and excellent insulating properties, PA6/GF composites have a wide range of applications in the aerospace industry [35].

Although PA6GF composites are widely popular in industry, their use in biomedicine is still in the development stage. PA6 is known for its excellent mechanical properties and resistance to flow, making it a potentially suitable material for biomedical applications. When combined with glass fibers (GFs), these composites can offer even better mechanical properties needed for various biomedical devices and applications, such as prosthetics, orthopedic implants, or surgical tools [25]. However, while PA6/GF composites provide excellent mechanical properties, their use in biomedicine is limited by the material’s biocompatibility. Therefore, it is important to continue testing and researching these materials to improve their safety and long-term stability in biological environments [38].

The challenges associated with using PA6GF composites in biomedicine primarily involve the biocompatibility of the material. While PA6 is relatively biocompatible, the addition of glass fibers may affect the material’s ability to interact with the human body. Additionally, the processing of PA6GF composites may involve the use of chemicals or additives that could influence their biocompatibility. Therefore, further research is needed to improve these materials to ensure they are safe and suitable for long-term use in the biomedical field [36].

## 5. Polyamide 12

Polyamide 12 (PA12) is one of the most widely used polymers in additive manufacturing technology, especially in powder bed fusion processes such as Multi Jet Fusion (MJF) and Selective Laser Sintering (SLS) [40]. These technologies enable the production of parts with high precision, low porosity, and the ability to tailor the geometry to the specific requirements of applications [41,42]. PA12, as a semi-crystalline polymer, has a broad processing window and excellent mechanical properties, including high strength, chemical resistance, and long-term durability [43,44]. These characteristics make it ideal for various applications, from the automotive and aerospace industries to medical devices [45].

Parts made from PA12 using MJF technology feature higher density and lower porosity compared to parts made with SLS, leading to better mechanical properties [42,46]. The manufacturing process in MJF also minimizes defects such as poorly fused particles and pores, further contributing to the reproducibility of mechanical properties [45]. Although MJF is a relatively new technology, it already offers significant advantages, including higher productivity and lower production costs while maintaining high product quality [41].

### 5.1. Mechanical Properties of PA12: Influence of Technology and Manufacturing Conditions

**Figure 13:**
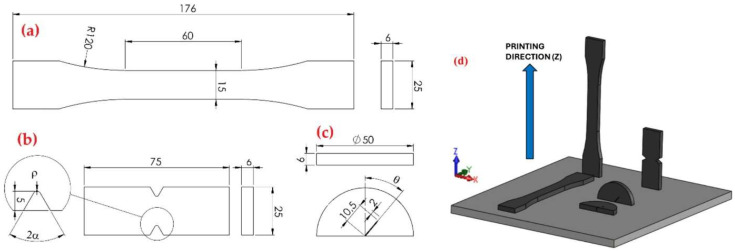
The image shows the dimensions and geometries of the test samples (dog bone, V-notch, semicircular bend) along with the orientation of their printing in the Z-direction for mechanical testing: (**a**) dog bone; (**b**) V-notched; (**c**) semi-sircular send; (**d**) reference system [41].

PA12, a technical semi-crystalline polymer, is widely used in additive manufacturing, primarily through technologies such as Multi Jet Fusion (MJF) and Selective Laser Sintering (SLS). Its mechanical properties are influenced by the manufacturing technology, process parameters, and print orientation. These factors are crucial for optimizing part performance for various industrial applications (Figure 13) [41].

#### 5.1.1. Anisotropy of Mechanical Properties

**Figure 14:**
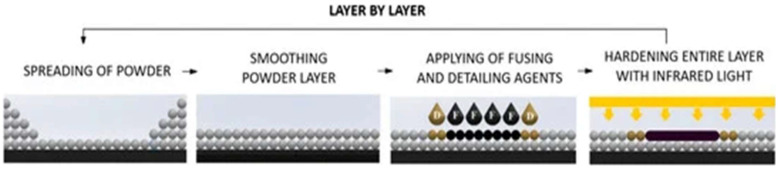
Schematic of the MJF (Multi Jet Fusion) manufacturing process [42].

The layered nature of parts produced using MJF and SLS methods causes mechanical anisotropy, with the print orientation significantly affecting tensile strength, elastic modulus, and fracture behavior (Figure 14). Research shows that parts printed along the Z-axis have weaker mechanical properties due to poorer bonding between layers [41,42]. In contrast, parts oriented horizontally exhibit better resistance to tensile loading and lower susceptibility to crack propagation [41,42].

MJF technology has demonstrated advantages over SLS, as it allows for more homogeneous sintering of layers and lower porosity. Andrea Avanzini et al. state that these properties lead to less variation in mechanical test results and greater repeatability of part performance [41].

#### 5.1.2. Geometric Discontinuities and Structural Integrity

PA12 parts with geometric discontinuities, such as notches or sharp edges, exhibit reduced strength and increased susceptibility to fracture. Experiments have shown that the sensitivity to notch opening angle decreases as the notch tip radius increases, with blunter notches having less impact on overall load-bearing capacity [41]. The integration of methods such as digital image correlation (DIC) and numerical simulations, such as finite element analysis (FEA), enables a detailed assessment of the mechanical behavior of notched components and prediction of their failure under load [41,42].

#### 5.1.3. Tribological and Fracture Properties

The mechanical properties of PA12, such as tensile strength and creep resistance, are influenced by the microstructure and layer arrangement. Studies confirm that parts produced using MJF technology have higher density and lower porosity compared to SLS parts, which results in better tribological properties and wear resistance [41,42,43].

The fracture mechanics of PA12 parts show that print orientation and loading conditions affect the type of fracture. In mixed loading modes (tension-shear), vertically printed parts exhibit higher susceptibility to brittle fracture, while horizontally oriented parts have greater resistance to ductile fractures. Analysis from Andrea Avanzini et al.’s study confirmed that configurations with larger notch radii improve the resistance of parts to mechanical stress [41].

#### 5.1.4. Sensitivity to Processing Conditions

Processing parameters, such as sintering temperature and layer formation, have a crucial impact on the final properties of PA12 parts. Optimizing the melt pool temperature in MJF helps reduce defects such as inadequate sintering or excessive porosity, improving the mechanical properties of the parts [42].

Andrea Avanzini et al. report that PA12 parts produced using MJF and SLS methods exhibit mechanical properties that depend on print orientation, processing parameters, and the presence of geometric discontinuities. Research confirms that MJF technology offers advantages in terms of better material homogeneity, lower porosity, and increased resistance to mechanical stress. However, to achieve consistent results, further investigation is required to assess the impact of processing parameters and defects on the final mechanical properties of PA12 parts [41].

**Figure 15:**
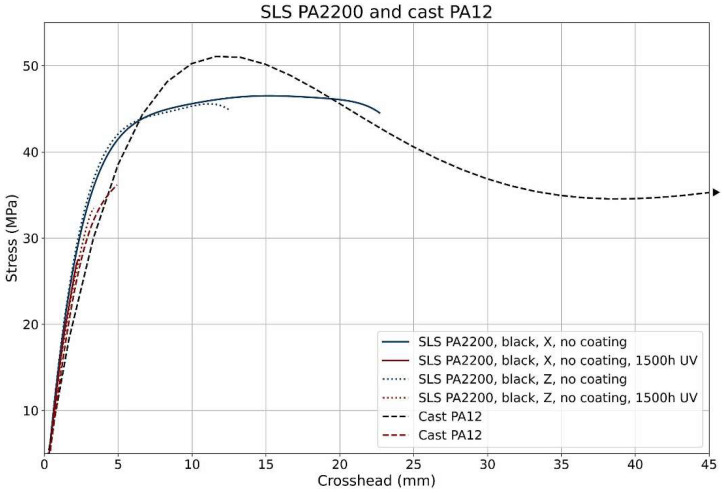
Stress vs. strain diagram with polynomial approximations for all conventionally manufactured materials. Samples without weathering influence are shown in black, while weathered samples are marked in red [19].

In Figure 15, the graph shows the dependence of stress on crosshead displacement in tensile testing for different PA12 polyamide samples, specifically for samples produced by selective laser sintering (SLS) and cast PA12 (cast PA12). The samples were subjected to different conditions, which is recorded in the legends of the graph [19].

### 5.2. Thermal and Crystallization Properties of PA12: Influence of Manufacturing Technology, Process Parameters, and Heat Treatment

Polyamide 12 (PA12) is a semi-crystalline polymer whose thermal and crystallization properties have a crucial impact on its processability and performance in various applications. These properties are influenced by manufacturing technology parameters as well as subsequent heat treatment. Understanding these aspects enables optimization of production and improvement of the functional properties of parts produced using additive manufacturing technologies [39,40].

#### 5.2.1. Thermal Properties and Their Significance in Processing

Polyamide 12 (PA12), which is an engineering semi-crystalline polymer that ensures good processability, hasa relatively high melting point of approximately 180 °C [41,42]. This allows for precise melting and sintering of powder particles, with differences in its principles affecting the thermal characteristics of the resulting parts. MJF uses an infrared heat source for uniform melting of entire layers, leading to homogeneous crystallization and reduced susceptibility to defects, while SLS uses a point laser that can cause local overheating and uneven crystallinity [42,43].

The thermal aging resistance and cycling of PA12 depend on the material’s microstructure. Research has shown that exposure to high temperatures above the glass transition temperature can lead to degradation of polymer chains, resulting in a reduction in strength and elasticity [44,45]. However, controlling processing parameters during production, such as melt temperature and cooling time, can improve thermal properties and ensure long-term stability [46].

Controlling cooling time during the production of PA12 products significantly impacts their thermal properties and long-term stability. Extended cooling times allow for more uniform crystallization, resulting in improved dimensional stability and enhanced mechanical strength. The slower cooling process reduces residual stresses within the material, which are often responsible for warping or deformation in the final product. Furthermore, controlled cooling facilitates the formation of a more ordered crystalline structure, improving the material’s resistance to thermal degradation over time. This relationship between cooling time and product properties underscores the importance of optimizing processing parameters to achieve superior performance and reliability in PA12-based applications [44].

#### 5.2.2. Crystallization Properties and Their Control

The crystalline structure of PA12 plays a crucial role in determining its mechanical and thermal properties. During the production of parts using MJF or SLS, rapid melting and subsequent cooling influence the size and distribution of crystallites [31,37]. Slow cooling results in the formation of larger crystals, which contribute to increased stiffness and thermal resistance of the parts, while rapid cooling leads to a finer microstructure that can enhance impact resistance [46].

One advantage of MJF is the ability to better control the cooling rate, which leads to more homogeneous crystallization throughout the part. In contrast, during SLS, layers are often exposed to uneven temperatures, which can cause variations in the crystallinity structure [44,46].

#### 5.2.3. Influence of Heat Treatment and the Relationship Between Thermal and Crystallization Properties

Annealing improves crystallinity by allowing the polymer to transition into more stable crystalline phases, such as the γ-phase or α-phase. The slow cooling during annealing enables better molecular alignment within the material, resulting in increased crystallinity and enhanced mechanical and thermal properties of the material [19].

Heat treatment after production can further optimize the properties of PA12 parts. Processes like annealing help remove internal stresses, increase crystallinity, and improve mechanical stability [19]. When properly selected temperature and time are applied, heat treatment can enhance the long-term resistance of parts to temperature cycles and mechanical stress [47].

Yunus Kutlu et al. highlight that despite the benefits of heat treatment, risks such as polymer degradation at excessively high temperatures must be considered. PA12 has a narrow processing window between its melting temperature (approximately 180 °C) and degradation temperature (around 320 °C), which requires precise control during heat treatment [44].

Porosity and anisotropy, which arise during layer-based manufacturing, directly affect thermal and mechanical properties. Controlling crystallinity through proper processing parameters and heat treatment allows for minimizing these drawbacks and achieving parts with improved performance characteristics [45].

The thermal and crystallization properties of PA12 are key factors affecting the quality of parts produced by additive manufacturing technologies [41,43,44,46].

## 6. Polyamide 12 Reinforced with Carbon Fiber

**Figure 16:**
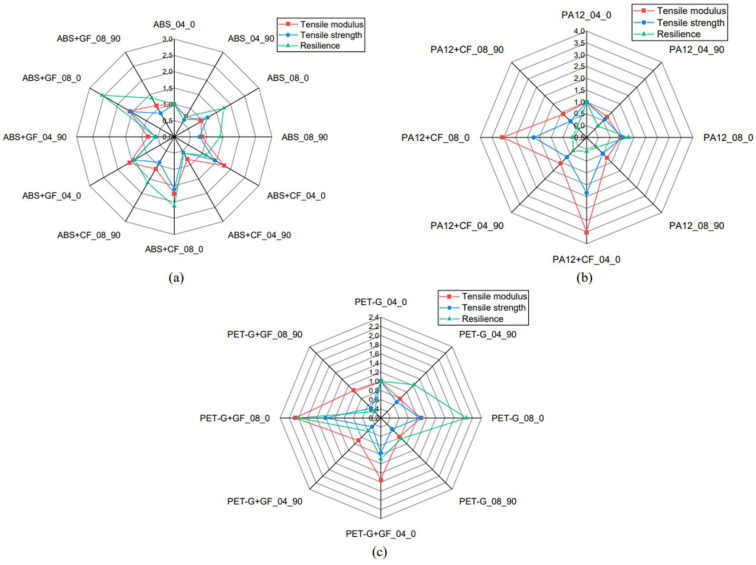
The diagram illustrates the mechanical properties of materials: (**a**) ABS; (**b**) PA12; (**c**) PET-G [45].

Polyamide 12 (PA12) is one of the thermoplastic polymers known for its excellent mechanical properties, flexibility, and impact resistance, making it a popular material for additive manufacturing, particularly with the use of Fused Filament Fabrication (FFF) [48]. The use of PA12 in 3D printing is very broad, ranging from biomedical manufacturing to the automotive and aerospace industries [49]. However, for specific applications requiring higher mechanical properties, such as strength, stiffness, and wear resistance, PA12 is often combined with reinforcing materials like carbon fibers to enhance its properties (Figure 16) [50].

Carbon fibers (CFs) are known for their excellent mechanical properties, such as high modulus of elasticity, tensile strength, and low weight, making this material ideal for use in composites for manufacturing functional and structural parts [51]. When combined with PA12, carbon fibers provide enhanced mechanical properties, including increased stiffness and strength, making PA12CF a highly attractive material for 3D printing and various applications that require an optimal combination of strength and low weight [52].

In recent years, research has intensely focused on studying the impact of different process parameters in 3D printing of PA12 reinforced with carbon fibers, according to Pejkowski, Ł., Seyda, J., Nowicki, K., and Mrozik, D. [44]. These experiments have shown that the addition of carbon fibers to PA12 brings significant improvements in mechanical properties, such as tensile strength, modulus of elasticity, and creep resistance, while maintaining the necessary flexibility and moldability for additive manufacturing [53]. These improvements are achieved through the optimization of process parameters, such as print speed, extrusion temperature, layer thickness, and print orientation [48].

This advancement in PA12CF has opened new possibilities for manufacturing complex, customized parts with high mechanical performance. The advantages of this technology are not limited to improving mechanical properties but also reducing costs and the time required to produce structures with complex geometries [49]. PA12CF composites have thus become a key material for a wide range of applications that require not only high strength and durability but also lightweight and manufacturing efficiency [50].

**Figure 17:**
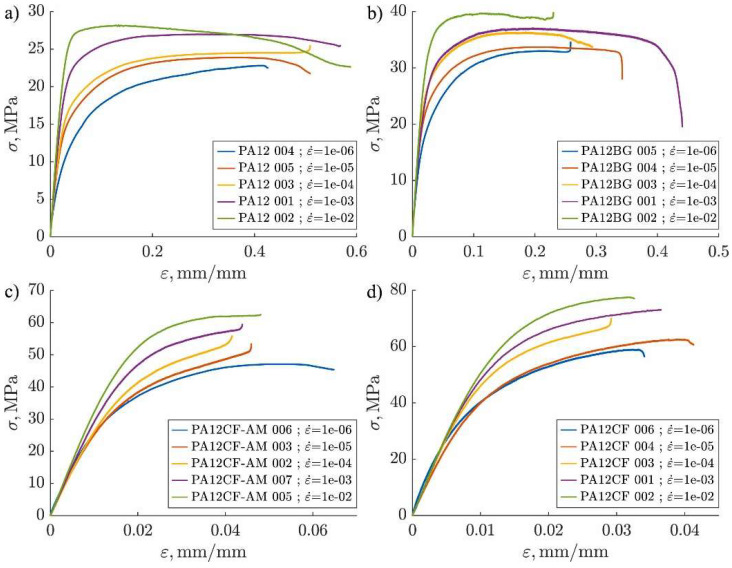
Monotonic stress–strain curves in tension for: (**a**) Z-NYLON™—polyamide 12 (PA12); (**b**) BubbleGlass™ (PA12BG); (**c**) NanoCarbon AM™ (PA12CF-AM); (**d**) NanoCarbon™ (PA12CF) [48].

In the context of the mechanical properties of carbon fiber-reinforced PA12, it is important to highlight the improvements that these composites provide compared to unreinforced polyamide. PA12, as a material, is known for its excellent mechanical properties, such as good impact resistance and high tensile strength; however, compared to carbon fiber-reinforced composites or other reinforcements, its mechanical properties are not optimal for applications requiring higher load-bearing capacity or resistance to fatigue processes (Figure 17). Experimental test results clearly confirm that the addition of carbon fibers significantly improves the material’s mechanical properties, especially tensile strength and modulus of elasticity. The tensile strength of PA12CF composites increased compared to unreinforced PA12, making this material an ideal candidate for demanding applications in the automotive, aerospace, and biomechanical industries, where high strength parameters and long-term material stability under constant or cyclic loading are required [48].

In addition to improving tensile strength, PA12CF composites also exhibit exceptional properties in terms of creep and viscoelastic behavior. Creep test results show that carbon fiber reinforcement positively impacts the material’s resistance to time-dependent deformations. While unreinforced PA12 exhibits significant deformation under constant load after only a few hours, PA12CF composites exhibit much lower deformation during creep, making them an ideal material for applications requiring high long-term stability under constant or cyclic loading [52]. Improved creep resistance is particularly important in applications where materials are exposed to long-term loading, such as components in the automotive industry, where materials are required to maintain their mechanical properties even after prolonged use [50].

**Figure 18:**
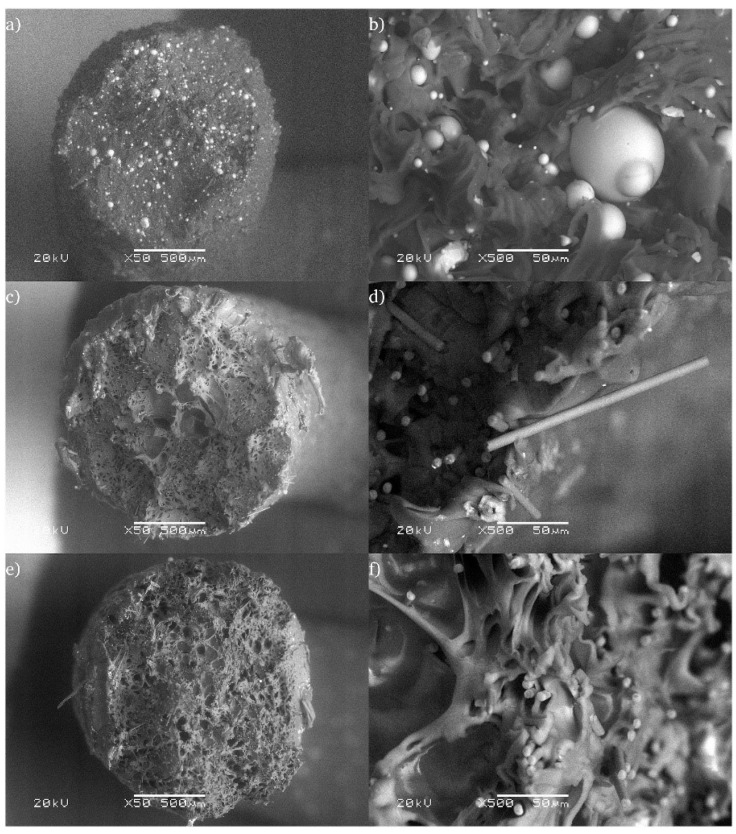
SEM images of cross-sections of the filaments used: (**a**,**b**) BubbleGlass™ (PA12BG); (**c**,**d**) NanoCarbon AM™ (PA12CF-AM); (**e**,**f**) NanoCarbon™ (PA12CF) [48].

An interesting aspect of PA12CF composite studies is the analysis of their microstructure and the effect of carbon fiber orientation on mechanical properties. SEM (Scanning Electron Microscopy) and CT (Computed Tomography) analyses revealed that the uniform distribution of carbon fibers in the PA12 matrix and their correct orientation during 3D printing contribute to a significant increase in the material’s mechanical properties. Carbon fibers oriented in the direction of the load provide higher strength and stiffness, supporting the material’s stability in demanding operating conditions. Furthermore, microscopic observations showed that proper composite processing, including optimal fiber orientation and correct 3D printing parameter settings, can significantly improve composite performance, reducing structural defects that could lead to material failure under high loads (Figure 18) [51].

The results of mechanical tests conducted at various strain rates confirm the impact of carbon fiber reinforcement on the dynamic behavior of PA12CF composites. As stated by Pejkowski, Ł. et al. the strain rate affects not only the tensile strength but also the fatigue resistance of the materials. The high tensile strength and tensile modulus achieved at different strain rates for PA12CF composites suggest that carbon fiber reinforcement provides materials not only with improved static mechanical properties but also better resistance to dynamic and cyclic loads [48].

Moreover, PA12CF composites demonstrate lower deformation values during creep compared to unreinforced PA12, indicating their ability to withstand long-term loads without performance degradation [53].

In applications where a combination of high mechanical properties and creep resistance is required, such as in the automotive industry or aerospace applications, PA12CF composites may offer an ideal solution. These materials show excellent stability under long-term loading while maintaining high tensile strength and modulus of elasticity, providing a wide range of potential applications in demanding industrial sectors [48].

### 6.1. Long-Term Durability of PA12CF Under Different Conditions

Polyamide 12 reinforced with carbon fibers (PA12CFs) has established itself in recent years as a material with excellent mechanical and thermal properties, finding broad applications in demanding additive manufacturing sectors. One of the key factors influencing material selection for specific applications is its long-term durability under various conditions. PA12CF demonstrates exceptional performance in terms of resistance to environmental factors such as temperature variations, humidity, mechanical stress, and time-dependent loads. These factors significantly impact the material’s behavior over time and directly influence its lifespan and reliability in high-demand applications [48].

One of the key aspects of the long-term durability of PA12CF is its resistance to creep. Due to the addition of carbon fibers, this material shows significantly better resistance to time-dependent deformations compared to unreinforced PA12. This behavior is particularly important in applications where the material is subjected to prolonged static or dynamic loads, such as automotive and aerospace components, which must maintain their strength and stability under constant loading over extended periods [40]. While unreinforced PA12 shows significant deformation after a few hours under constant load, as reported by Rodríguez-Reyna et al., PA12CF maintains its structural integrity for many hours and days, making it ideal for demanding applications requiring long-term stability [50].

Another factor contributing to the long-term durability of PA12CF is its exceptional ability to resist extreme temperatures and humidity. This material shows minimal degradation of mechanical properties when tested under extreme conditions, such as high temperatures and increased humidity. Compared to other materials, PA12CF remains stable even under various environmental changes, making it an ideal material for applications in various industrial sectors that must withstand temperature and humidity variations, such as automotive parts, aerospace components, or industrial machines and equipment [51].

Experimental test results also demonstrate that PA12CF exhibits excellent wear resistance. Thanks to the combination of the outstanding mechanical properties of carbon fibers and the thermoplastic matrix of PA12, this composite can maintain its structure even under prolonged exposure to repeated mechanical loads. This is especially important in cases where components are subjected to continuous mechanical loading or vibrations, such as in the automotive or aerospace industries [25]. In these sectors, where high reliability and minimal material wear are required, PA12CF is the perfect choice for producing critical components that must endure harsh conditions for long periods [53].

PA12CF thus proves to be a material with outstanding long-term durability under various operational conditions. Its ability to withstand deformations, creep, temperature and humidity changes, as well as mechanical wear, makes it an ideal candidate for applications requiring a combination of long-term stability, high mechanical properties, and resistance to demanding conditions. This material can maintain its optimal properties over a long period, making it an excellent material for a wide range of industrial applications [53].

### 6.2. Applications of PA12CF in Biomedicine, Healthcare, and Industry

Carbon fiber-reinforced polyamide 12 (PA12CF) has shown promise as a material in a wide range of applications in biomedicine, healthcare, as well as in the automotive and aerospace industries. With its excellent mechanical properties such as high tensile strength, stiffness, and wear resistance, these composites are an ideal solution for the production of various components that must meet demanding operational conditions [48].

#### Applications in Biomedicine and Healthcare

In biomedical applications, PA12CF is primarily used in the production of customized implants and prosthetics that require a combination of high mechanical properties and biological compatibility. The carbon fibers in PA12CF enhance the material’s stability, ensuring long-term resistance and robustness, which is crucial in environments where long-term integration with the human body is needed. PA12CF is thus used in various prosthetic applications, such as foot, hand, joint prostheses, and other body parts, where there is a demand for a material that provides similar mechanical properties to natural bones and tissues [49].

PA12CF-based composites also hold potential in the production of microfluidic chips and other biomedical devices, which are often used in diagnostics or research. Their advantage lies not only in mechanical strength but also in their ability to be 3D printed with high precision and tailored to the individual needs of the patient. In the biomedical field, it is essential to ensure the bioacceptability and compatibility of the materials, paving the way for further research and development [50].

### 6.3. Impact of 3D Printing Process Parameters on PA12CF Properties

Print Speed

Print speed significantly affects the mechanical properties of PA12CF. At higher print speeds, insufficient heating of the material can occur, which affects the adhesion between layers and subsequently reduces the strength and stiffness of the composite. Experiments have shown that optimizing print speed improves the microstructure and mechanical durability of the material [48].

Extrusion Temperature

Extrusion temperature is crucial for the quality and properties of the printed material. Low extrusion temperatures can lead to incomplete melting of the material, affecting the mechanical strength and stability of the composite. On the other hand, excessively high temperatures can cause material degradation. Therefore, optimizing the extrusion temperature is essential to achieve the desired mechanical properties [51].

Print Orientation

Print orientation can significantly influence the mechanical properties of PA12CF. The distribution of layers and the orientation of the carbon fibers are key factors affecting the strength and stiffness of the material. Proper orientation, with fibers aligned in the direction of the load, provides significantly higher mechanical properties, which increases the material’s resistance under demanding conditions [52].

Layer Thickness

Layer thickness also plays an important role in 3D printing of PA12CF. Thinner layers can improve print resolution and the stability of details but may reduce tensile strength. Optimal layer thickness ensures a balance between detail and mechanical properties, leading to optimal quality of the composite [53].

## 7. Polyamide 12 Reinforced with Glass Fiber

**Figure 19:**
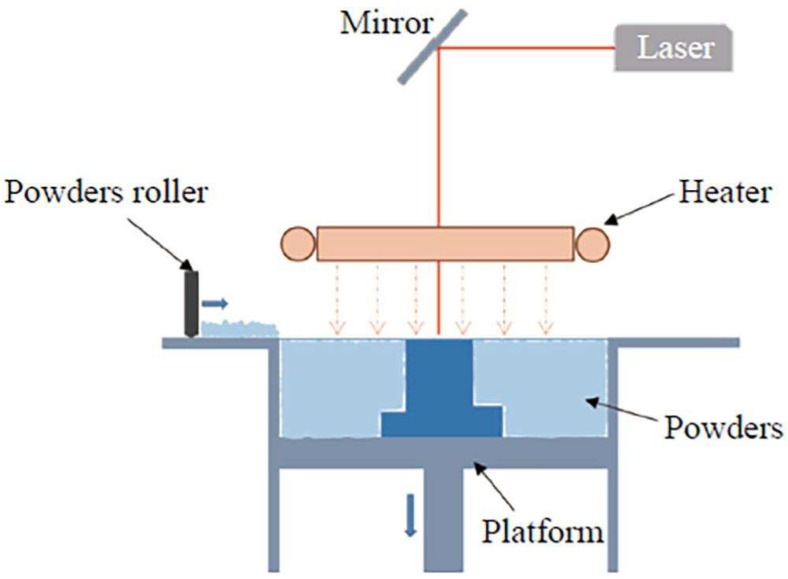
Schematic of Selective Laser Sintering (SLS). Reproduced with permission from [54], Elsevier, 2025.

Selective Laser Sintering (SLS) is a rapid prototyping technology where 3D objects are created by fusing powdered materials layer by layer using infrared laser beams (Figure 19). This process offers several advantages, such as a wide selection of materials, high precision, the ability to produce complex components and molds without the need for support, and high material utilization. Polyamide 12 (PA12) is an ideal material for this technology due to its low processing temperatures, low laser power requirements, and high precision. However, 3D-printed PA12 parts often exhibit lower mechanical properties compared to conventionally molded parts, which is due to the presence of small porous areas in the material [54].

In recent years, there has been a significant increase in research on 3D-printed composites, particularly those containing fibers in PA12, aimed at improving electrical conductivity and mechanical stiffness. Various studies have focused on the tensile properties and failure behavior of fiber-reinforced composites, including carbon and glass fibers. For example, some research has investigated the effect of temperature on the fracture behavior of PA12 and PA12 reinforced with glass fibers processed using SLS. These studies have shown that the presence of glass fibers significantly improves the tensile strength and elasticity of PA12 composites, enhancing their mechanical properties during deformation [55].

### 7.1. Mechanical Properties of PA12GF

Various types of fibers, such as carbon and glass fibers, have a significant impact on the mechanical properties of PA12 composites. Glass fibers have proven to be effective in enhancing the tensile strength and stiffness of PA12, making these composites highly suitable for applications requiring high mechanical performance. These materials exhibit excellent energy absorption and can withstand repeated loading without significant deformation. However, while the addition of glass fibers improves mechanical properties, it may also lead to a reduction in the thermal stability of the composite during processing [56].

The reason for the improvement lies in the presence of glass fibers, which significantly enhance the ability of PA12 composites to better absorb energy and resist mechanical damage under repetitive loading. Glass fibers improve wear resistance and fatigue performance, which is particularly important in applications where higher wear resistance and fatigue performance are required.

This effect is due to the fact that glass fibers increase the strength and stability of the composite, allowing it to better withstand mechanical stresses, especially in environments where long-term resistance to repeated mechanical impacts or fatigue is required [56].

In addition to improving mechanical properties, the integration of glass fibers into PA12 composites can also affect the material’s thermal properties. Studies have shown that adding fibers can enhance the thermal stability of the composite while improving its resistance to thermal degradation during processing. This is especially important in applications where the composite is exposed to high temperatures or thermal cycles [57].

A recent study by A.J. Cano, A. et al., which examined the mechanical properties and failure modes of 3D-printed lattice structures from PA12 and glass fiber/PA composites using digital image correlation (DIC), revealed that adding glass fibers significantly increased the tensile strength, with the tensile strength of the samples increasing by 17.2% and the elasticity modulus increasing by 82.01%. These structures also exhibited good energy absorption capabilities in compression and withstood repeated loading without significant deformation [58].

The development of multifunctional composite materials, which combine structural and thermal energy storage (TES) capabilities, has become another significant goal in the field of polymer composites. This is achieved by integrating phase change materials (PCMs) into polymer matrices such as PA12. The ability to store and release thermal energy is critical for various applications such as thermal management in buildings, electronic devices, and automotive components. Incorporating PCMs into PA12 composites enhances their mechanical properties while allowing them to perform TES functions [51].

In recent years, research into PA12 composites reinforced with glass fibers has increasingly focused on their energy absorption capabilities and resistance to repeated loading. Three-dimensional-printed lattice structures from PA12 and composites with added glass fibers have been the subject of research by Wenfeng Hao, Ye Liu, Tao Wang, Guangping Guo, Haosen Chen and Daining Fang, who used digital image correlation (DIC) to analyze deformation and stress fields during tensile and compression tests. These experiments demonstrated that the presence of glass fibers significantly improves mechanical properties such as tensile strength and elasticity, with the tensile strength of the samples increasing by 17.2%, and the elasticity modulus of the samples increasing by 82.01%. In compression, these composites showed excellent energy absorption capability and demonstrated high resistance to repeated loading, making them key for applications in various industrial sectors such as automotive and construction [54].

In addition to mechanical properties, PA12 composites with glass fibers also affect the thermal stability of the materials. Research has shown that the presence of glass fibers in PA12 improves their resistance to thermal degradation during processing, which is particularly important for applications where these materials are exposed to high temperatures or thermal cycles. The improvement in thermal stability results from the combination of glass fibers with the thermoplastic matrix, leading to better thermal breakdown management and increasing the long-term performance of the material [55].

### 7.2. The Structure of PA12GF

Other important properties influenced by the presence of glass fibers in PA12 include the performance of lattice structures. Studies have focused on the impact of these composites on performance under different temperatures and types of loading. Some experiments have shown that lattice structures made from PA12 composites with glass fibers exhibit better energy absorption and resistance to mechanical damage under repetitive loading compared to composites without glass fibers. This effect is particularly significant in applications where higher wear resistance and fatigue performance are required [57].

Research by Chunze Yan et al., focusing on the mechanical properties of PA12 composites reinforced with glass fibers, has shown that these materials exhibit significant improvements compared to pure PA12. In addition to improved tensile strength and elasticity, the addition of glass fibers allowed for better resistance to fracture and cracking. Particularly interesting are the results regarding the ability of these composites to absorb energy at different temperatures, indicating their potential use in a wide range of applications, from the automotive industry to aerospace [57].

The growing interest in PA12 composites reinforced with glass fibers is a result of their excellent mechanical and thermal properties, making them ideal for critical applications where performance and reliability requirements are high. Furthermore, research by A.J. Cano, A. Salazar, and J. Rodríguez indicates that the combination of PA12 and glass fibers provides materials capable of effectively storing thermal energy while also offering the required mechanical strength for demanding industrial applications [58].

PA12 composites reinforced with glass fibers, produced using selective laser sintering (SLS) technology, have become highly promising materials in various industrial sectors in recent years. The advantages offered by SLS—such as high precision, the ability to create complex shapes, and the use of a wide range of materials—make these composites attractive for applications requiring a combination of mechanical strength and heat resistance. PA12 composites reinforced with glass fibers are particularly valued in applications where excellent tensile strength and stability at various temperatures are needed [54].

### 7.3. Industrial Applications of PA12GF

One of the key areas where PA12 glass fiber-reinforced composites are used is the automotive industry. In this field, materials are required to be strong, lightweight, and impact-resistant. The glass fibers added to PA12 not only improve the mechanical strength of the materials but also their ability to absorb energy during impacts, which is essential for protection in case of accidents. These composites are often used to manufacture components such as bumpers, load-bearing frames, or interior parts of vehicles, which must meet strict safety and durability standards [54].

The aerospace industry is another area where PA12 composites have great potential. Aircraft components such as engine covers, mounting parts, and cabin equipment structures require materials that must not only be strong and resistant to high temperatures but also lightweight in order to reduce the overall weight of the aircraft. PA12 glass fiber-reinforced composites meet these requirements and are used in various aerospace applications where a combination of mechanical strength and resistance to thermal degradation is needed. Due to their thermal and mechanical properties, they have become a key material in the aerospace industry, where the performance requirements for materials continue to grow [55].

In construction and heavy industry component manufacturing, PA12 composites are also crucial. These composites are used to manufacture structures and support components that must withstand harsh external conditions. They have found their place in applications such as the production of prefabricated components, construction molds, or components exposed to chemical effects or moisture. Due to their enhanced mechanical properties, such as impact resistance and corrosion resistance, PA12 glass fiber-reinforced composites ensure long service life and low maintenance requirements in extreme conditions [56,57].

In addition to these industries, PA12 composites are also used in electronics, particularly for the manufacture of components that must meet strict requirements for thermal and electrical conductivity. In this field, the glass fibers in PA12 composites are especially valued for their ability to improve electrical insulation and mechanical stability in electronic devices, such as protectors, covers, or component holders exposed to high temperatures [58].

PA12 glass fiber-reinforced composites not only feature excellent mechanical properties but also offer significant potential in areas where thermal energy management capabilities are required. This phenomenon can be achieved by combining PA12 with phase change materials (PCMs) that enable the material to effectively store and release heat. Such materials find applications in scenarios where thermal changes need to be managed and stable thermal conditions maintained during use. G. Fredi et al. studied how PA12 composites combined with PCM can perform these functions, making them suitable for solar and thermal applications [55].

In the field of smart thermal management, PA12 composites combined with PCM are used in various applications, such as smart textiles and thermal management in electronic devices. These composites can effectively absorb excess heat during operation and then release it when the temperature drops. In electronic devices such as mobile phones or computer components, which generate a significant amount of heat during use, these materials contribute to efficient heat dissipation, improving performance and extending lifespan. Cano et al. highlight the ability of PA12 composites to enhance thermal properties at different temperatures, which is crucial for electronics [50]. The combination of PA12 and PCM also allows the creation of materials with adjustable thermal properties, making them ideal for applications where thermal conditions must be tailored to specific requirements.

Interesting applications of PA12 glass fiber-reinforced composites with PCM are also found in solar thermal systems. In these systems, materials are capable of capturing heat during sunny days and storing it for later use. Thanks to the ability of PA12 composites with PCM to perform these functions, we can efficiently utilize solar energy, reducing energy costs and improving the ecological properties of buildings and devices. Combined with the properties of glass fibers, which enhance mechanical stability and durability, these composites are ideal for use in buildings and solar thermal systems where stability and long-term reliability of materials are required [54].

## 8. Future Development of PA6 and PA12

Polyamide 6 (PA6) and polyamide 12 (PA12) possess distinct properties that make them suitable for specific applications. PA12 excels in low moisture absorption, chemical resistance, and dimensional stability, whereas PA6 offers higher tensile strength and elastic modulus, making it essential for mechanically demanding applications.

PA12 composites are produced using modern technologies such as SLS and MJF, which provide precise control over part fabrication. PA6 composites are often processed using traditional methods like extrusion, though FDM technology improves filler distribution and reduces moisture absorption.

PA12 is utilized where chemical resistance and stability are critical, while PA6 is preferred for applications requiring high mechanical strength. The choice of material depends on the specific requirements of the application.

The future development of PA6 and PA12 offers opportunities to improve their properties and expand applications across various industrial sectors. Areas of focus for future innovations include enhancing mechanical properties, optimizing manufacturing processes, and developing sustainable materials to meet growing industrial demands.

- Improvement of Mechanical Properties

Increased tensile strength: The goal is to achieve tensile strength values exceeding 90 MPa (PA6) and 95 MPa (PA12), enabling the use of these materials in high-stress applications such as automotive parts and structural components.

Higher modulus of elasticity: Increasing the modulus of elasticity above 2.5 GPa (PA6) and 3.0 GPa (PA12) will ensure their suitability for applications requiring high stiffness, such as in engineering and robotics.

Improved creep resistance: Development of PA6 and PA12 with better resistance to long-term loading, extending their lifespan in dynamic and static applications.

- Resistance to Extreme Conditions

Wider temperature range: Ensuring stable mechanical properties within the range of −40 °C to 150 °C for PA6. One of the key areas of future development for PA12 is improving its dimensional stability at elevated temperatures, particularly near its melting point of 180 °C. Although PA12 begins to melt at this temperature, current research is focused on enhancing its thermal stability through advanced processing techniques and material modifications. The challenge lies in maintaining mechanical properties and dimensional integrity at high temperatures, which is crucial for applications in demanding environments such as aerospace and energy. To address this issue, future development will likely involve the use of specialized stabilizers, fillers, and advanced heat treatment processes that will allow PA12 to better withstand these temperatures without compromising its mechanical properties.

Improved moisture resistance: Development of materials with lower moisture absorption, which will improve their dimensional stability and mechanical properties in high-humidity environments.

Enhanced properties at cryogenic temperatures: Optimization of PA6 and PA12 properties for applications at temperatures near absolute zero, minimizing material brittleness.

- Optimization of Manufacturing Processes

Better material homogeneity: Use of nano-additives to control the homogeneity of the structure and reduce internal stresses by 20%.

Adaptive manufacturing methods: Implementation of processes that allow precise adjustment of properties according to the requirements of specific applications.

Advanced processing technologies: Utilization of innovative technologies such as laser processing to minimize production time without compromising quality.

- Sustainability and New Materials

Ecological alternatives: Increasing the market share of biologically derived PA6 and PA12 to at least 30% by 2030.

Durability of ecological variants: Development of materials resistant to UV radiation, moisture, and chemicals to achieve parameters comparable to traditional variants.

Nanotechnology: Integration of nanoparticles to enhance mechanical and thermal properties, opening new possibilities in high-tech applications.

## 9. Conclusions

Polyamide materials, such as PA6 and PA12, represent one of the most essential pillars of modern technologies due to their exceptional combination of properties. Their high strength, resistance to chemical influences, low weight, and thermal stability make them ideal for a wide range of industrial sectors, from the automotive and aerospace industries to energy and electronics. Research and development in this field have brought significant improvements to their properties, further strengthening their application potential.

Mechanical properties, such as tensile strength, modulus of elasticity, and wear resistance, have been significantly enhanced through innovative technologies and the addition of various reinforcing materials. Today, PA6 and PA12 can withstand not only high mechanical loads but also demanding environmental conditions, making them indispensable materials for modern engineering.

Research highlights several key areas where these materials are being optimized:

Improved resistance to moisture and chemicals: Thanks to chemical modifications and protective surface treatments, water absorption is minimized, improving dimensional stability and the lifespan of materials, particularly in environments with high humidity or aggressive chemicals.

Increased thermal stability: The addition of ceramic fillers and modified resins allows PA6 and PA12 to withstand high temperatures and thermal degradation, expanding their use in environments with extreme temperature fluctuations.

Mechanical enhancements: The integration of reinforcing materials, such as carbon and glass fibers, significantly increases tensile strength, modulus of elasticity, and resistance to delamination, which is particularly important for applications requiring high structural integrity.

While some authors consider the processing window of PA12 to be wider in the context of its versatility in processing (e.g., under standard processing conditions), others argue that it is narrower when considering the need for precise temperature control to prevent material degradation, especially in applications that require high reliability and mechanical properties. Therefore, the processing window of PA12 can be defined as either wider or narrower, depending on the specific processing conditions and application requirements.

Innovative surface treatments: Surface treatments based on chemical and hybrid processes significantly improve adhesion between the polyamide matrix and reinforcing fillers, enhancing the efficiency of stress transfer and overall mechanical strength.

Eco-friendly approaches: With increasing pressure for sustainability, emphasis is placed on developing bio-based polyamides and using eco-friendly additives, reducing the environmental impact while maintaining or even improving the technical properties of materials.

Hybridization and multifunctional properties: The deployment of nanotechnologies, such as carbon nanotubes or oxidized graphene, opens new possibilities for PA6 and PA12. These materials gain unique properties, such as electrical conductivity, self-healing capabilities, or increased thermal conductivity, thereby expanding their application in specialized fields.

These advancements not only push the boundaries of PA6 and PA12 materials’ possibilities but also open new paths for their use in demanding and innovative applications. In environments requiring extreme strength, temperature resistance, or chemical resilience, PA6 and PA12 prove to be indispensable solutions. Their ongoing development also contributes to meeting environmental goals, making them key materials for the sustainable future of industrial production.

## Data Availability

Not applicable.
